# Taxonomic and Functional Dysregulation in Salivary Microbiomes During Oral Carcinogenesis

**DOI:** 10.3389/fcimb.2021.663068

**Published:** 2021-09-16

**Authors:** Jiung-Wen Chen, Jer-Horng Wu, Wei-Fan Chiang, Yuh-Ling Chen, Wei-Sheng Wu, Li-Wha Wu

**Affiliations:** ^1^Department of Environmental Engineering, National Cheng Kung University, Tainan, Taiwan; ^2^Department of Oral & Maxillofacial Surgery, Chi-Mei Medical Center, Liouying, Taiwan; ^3^School of Dentistry, National Yang-Ming University, Taipei, Taiwan; ^4^Institute of Oral Medicine, College of Medicine, National Cheng Kung University, Tainan, Taiwan; ^5^Department of Electrical Engineering, National Cheng Kung University, Tainan, Taiwan; ^6^Institute of Molecular Medicine, College of Medicine, National Cheng Kung University, Tainan, Taiwan

**Keywords:** oral cancer, microbiome dysbiosis, machine learning, oral verrucous hyperplasia, saliva

## Abstract

Exploring microbial community compositions in humans with healthy versus diseased states is crucial to understand the microbe-host interplay associated with the disease progression. Although the relationship between oral cancer and microbiome was previously established, it remained controversial, and yet the ecological characteristics and their responses to oral carcinogenesis have not been well studied. Here, using the bacterial 16S rRNA gene amplicon sequencing along with the *in silico* function analysis by PICRUSt2 (Phylogenetic Investigation of Communities by Reconstruction of Unobserved States 2), we systematically characterized the compositions and the ecological drivers of saliva microbiome in the cohorts of orally healthy, non-recurrent oral verrucous hyperplasia (a pre-cancer lesion), and oral verrucous hyperplasia–associated oral cancer at taxonomic and function levels, and compared them with the re-analysis of publicly available datasets. Diversity analyses showed that microbiome dysbiosis in saliva was significantly linked to oral health status. As oral health deteriorated, the number of core species declined, and metabolic pathways predicted by PICRUSt2 were dysregulated. Partitioned beta-diversity revealed an extremely high species turnover but low function turnover. Functional beta-diversity in saliva microbiome shifted from turnover to nestedness during oral carcinogenesis, which was not observed at taxonomic levels. Correspondingly, the quantitative analysis of stochasticity ratios showed that drivers of microbial composition and functional gene content of saliva microbiomes were primarily governed by the stochastic processes, yet the driver of functional gene content shifted toward deterministic processes as oral cancer developed. Re-analysis of publicly accessible datasets supported not only the distinctive family taxa of *Veillonellaceae* and *Actinomycetaceae* present in normal cohorts but also that *Flavobacteriaceae* and *Peptostreptococcaceae* as well as the dysregulated metabolic pathways of nucleotides, amino acids, fatty acids, and cell structure were related to oral cancer. Using predicted functional profiles to elucidate the correlations to the oral health status shows superior performance than using taxonomic data among different studies. These findings advance our understanding of the oral ecosystem in relation to oral carcinogenesis and provide a new direction to the development of microbiome-based tools to study the interplay of the oral microbiome, metabolites, and host health.

## 1 Introduction

Oral cavity is a dynamic and complex ecosystem, harboring more than 1,000 species of microorganisms (Lamont et al., 2018). The ecological balance of the host-microbiome symbiosis benefits human health by supporting the host immune system, maintaining physiological functions, and providing additional metabolic potentials (Kilian et al., 2016) to inhibit the growth of exogenous/opportunistic pathogens (Kreth et al., 2005; Wescombe et al., 2009) and regulate the host-microbe homeostasis such as systemic nitrate metabolism that is linked to cardiovascular diseases (Govoni et al., 2008; Farah et al., 2018). Recent studies have shown that the oral microbiome plays an essential role in the etiology of oral and systemic diseases, such as caries, periodontitis, and oral cancer (Socransky and Haffajee, 2005; Costalonga and Herzberg, 2014; Gao et al., 2018). Among these diseases, oral cancer is of particular concern because it causes approximately 180,000 deaths a year worldwide (Ferlay et al., 2019). Oral bacteria, along with other known risk factors (smoking, alcohol, and betel quid chewing), have been reported to be associated with oral cancers (Katz et al., 2011; Lin et al., 2011; Zhao et al., 2017). Oral carcinogenesis has been considered a pivotal factor to alter the oral microbiome, while the microbiome dysbiosis may exacerbate the disease progression in the host. For example, using the gnotobiotic mouse model of oral cancer, research demonstrated that the oral microbiome regulated a specific signaling pathway to promote tumorigenesis in oral cancer (Stashenko et al., 2019). A recent study further provides causal evidence in promoting oral tumorigenesis *via* crosstalk between signaling pathways by periodontal pathogens (Kamarajan et al., 2020). Although the relationship between microbiome and cancer is still controversial and complicated (Sepich-Poore et al., 2021), these studies have pointed out the complex mutual interplay between the oral microbiome and oral carcinogenesis.

Oral potentially malignant disorders (OPMDs) describe a diverse group of lesions or conditions, including leukoplakia, erythroplakia, oral submucous fibrosis, and oral verrucous hyperplasia (OVH), that may precede the development of oral squamous cell carcinoma (OSCC), accounting for more than 90% of oral cancers (Markopoulos, 2012; Warnakulasuriya, 2020). Though oral cavity can be easily accessed for oral cancer screening, more than 60% of patients were detected at a late stage of OSCC partly due to the unawareness of patients and healthcare practitioners for the asymptomatic lesions (Mashberg, 2000; Mager et al., 2005; Lingen et al., 2008; Kaur et al., 2018). Conventional oral examination (COE) followed by confirmatory tissue biopsy is the gold standard for oral cancer diagnosis (Lingen et al., 2008). However, COE may not be able to identify all OPMD lesions or lesions that are prone to progress to OSCC (Lingen et al., 2008). Besides, the tissue biopsy is invasive, painful, and time-consuming (Kaur et al., 2018). Although other clinical diagnostic tools were available for oral cancer detection (Mashberg, 1983; Eisen, 2002), patients are still diagnosed in the late stages of OSCC (Kaur et al., 2018). Therefore, early detection and diagnosis technology for OPMD and oral cancer are necessary, and saliva serves as an ideal reservoir for non-invasive biomarker exploration.

Studies have suggested that the oral microbiome changed during the progression of OSCC (Zhao et al., 2017; Hashimoto et al., 2019; Chen et al., 2020). These studies mainly focused on two cohorts (healthy control/OSCC or OPMD/OSCC) instead of three (healthy control/OPMD/OSCC) without a follow-up.

However, the progress of OPMD malignancy usually takes years to more than decades, with malignant transformation rates ranging from less than 1% to over 30% (Chiang et al., 2020; Warnakulasuriya, 2020). Although a few oral microbiome studies did include OPMD samples (Hernandez et al., 2017; Lee et al., 2017), the inclusion of a wide variety of OPMDs may confound the results. Among OPMDs, OVH is commonly detected in the oral cavity of betel quid chewers and has high transformation rates of up to 21.3% (Chiang et al., 2020; Warnakulasuriya, 2020). So far, the interaction between the oral microbiome and OVH carcinogenesis has not yet been reported.

The microbial community structure of the oral cavity remains compositionally stable to ecological determinants (e.g., pH, redox, and nutrients) due to its capability of resistance and resilience (Richards et al., 2017; Marsh, 2018). The stability may be substantially perturbed by stressors, driving the microbial communities into distinct patterns in taxonomical and functional components (Zaneveld et al., 2017), and this concept was captured in several hypotheses regarding the microbial ecology and oral diseases. For example, the ecological plaque hypothesis postulated that caries and periodontal diseases are a consequence of a taxonomic profile change of plaque microbiota driven by an altered environment (Marsh, 2003). For the host-microbiome ecosystem of OSCC, the oral microbiome may initially comprise species with competitive advantage *via* host selection, followed by a functional dysbiosis and enhancement of OSCC development as virulence factors of selected microbes are expressed (Al-Hebshi et al., 2019). To decipher how microbiomes respond to stressors, the patterns of microbial composition and the underlying ecological drivers were usually studied using taxonomic data. A recent systemic review of studies on the microbiome of OSCC patients reported that the tumor-associated microbiome presented similar functional potentials regardless of variations in taxonomic profiles (Al-Hebshi et al., 2019). Thus, taxonomic information in conjugation with functional profiles may shed some light on the variation of the oral microbiome.

The between-sample diversity (beta diversity) is often used to measure the differences between samples and can be disentangled into nestedness and turnover components: the former is a non-random process of species loss or gain, while the latter is the replacement of some species by others (Baselga, 2010). These patterns are microbial responses to deterministic processes, stochastic processes, or combinations of the two (Chase and Myers, 2011; Stegen et al., 2013; Zhou and Ning, 2017). As such, the quantitative determination of the ecological drivers influencing community composition in an ecosystem is important for explicitly elucidating the community dynamics. By quantifying the stochasticity ratio using the pattern-oriented null model (Ning et al., 2019), our previous study showed the dominance of stochastic perturbations in shaping the oral microbiomes of oral submucous fibrosis (one of the OPMDs) and OSCC cohorts (Chen et al., 2020). The influence of stochastic perturbations might also be crucial in the healthy group, given that the oral microbiome was highly personalized and time-varied (Mukherjee et al., 2018). Since highly diverse microorganisms would survive in similar ecosystems (i.e., oral cavity of healthy individuals, OPMD, and OSCC cohorts), we, therefore, were interested in exploring whether the disease stressor can shape the functional gene content of oral microbiome and the functional dysbiosis would occur in response to the development of oral carcinogenesis.

In the present study, we hypothesized that the alteration of oral microbiomes of orally healthy (normal), OPMD (specifically OVH), and OSCC cohorts were associated with oral health status. To investigate the role of ecological patterns in healthy and diseased oral microbiomes, both taxonomic profiles and functional potentials were studied in terms of the dichotomy of beta diversity (nestedness and turnover), along with the stochasticity ratio. To our knowledge, this is the first report to disentangle the contribution of the turnover and nestedness of both taxonomic and functional compositions in three different states of the oral cavity ecosystem (orally healthy, OPMD, and OSCC). We further validated our results with publicly available data using the same pipeline and the machine learning prediction.

## 2 Materials and Methods

### 2.1 Study Participants and Sample Collection

All participants were recruited from Chi Mei Medical Center (CMMC), Liouying, Taiwan, with the approval of the Institutional Review Board of CMMC (IRB No.: 10612-L02). Participants were interviewed to ensure no antibiotics or surgical treatments for at least one month to enrollment and instructed to refrain from eating, drinking, or using oral hygiene products for at least one hour prior to saliva collection and to rinse their mouth with drinking water. Five minutes after oral rinsing, participants were instructed to spit into a 50 mL centrifuge tube, which was kept on ice, and were cautioned not to cough up sputum. A total of 5 mL of saliva was collected from each participant within a 30-minute time frame. Saliva samples were then centrifuged at 2,600 × *g* at 4°C for 15 min. One milliliter of the supernatant was transferred to a new centrifuge tube for other research, and the rest of the saliva supernatant was treated with RNase Inhibitor (Ambion, Austin, TX, USA) and stored at −80°C for further analysis. The samples were processed and frozen within 30 minutes after collection.

### 2.2 DNA Extraction, PCR, and 16S rRNA Gene Sequencing

Bacterial genomic DNA was extracted from saliva samples using a QIAamp DNA Mini Kit (Qiagen, Germany) according to the manufacturer’s spin column protocol. The extracted DNA was amplified using a barcoded *Bacteria*-specific primer set (341F/806R) that targets the V3–V4 hypervariable region of the 16S rRNA gene. The PCR amplicons were sequenced on a MiSeq platform (Illumina, USA) using v3 Chemistry Kits (2 × 300 bp). The detailed sequencing protocol has been described previously (Chen et al., 2017).

### 2.3 Bioinformatics Analyses

#### 2.3.1 16S rRNA Sequence Processing

High-throughput amplicon sequencing data were analyzed on the QIIME 2 platform (v2019.4) (Bolyen et al., 2019). After the primers at both ends were trimmed, raw sequences were quality filtered, denoised, merged, and chimera filtered using DADA2 to produce ASVs (Callahan et al., 2016), which provides a finer resolution of sequence variants down to single nucleotide differences compared to traditional 97% similarity of operational taxonomic units. The maximum number of expected errors was set at 3. The denoised ASVs with lengths outside the interval between 400 and 450 nt were excluded from the subsequent analysis. To obtain taxonomy at the species level with a focus on bacteria present in oral cavity, taxonomic annotation of ASVs was performed by a customized naïve Bayes classifier trained on the expanded Human Oral Microbiome Database (version 15.1) (Escapa et al., 2018) using the q2-feature-classifier plugin (Bokulich et al., 2018b) with default settings. “Unclassified” was appended to the lowest available taxonomic level for ASVs that were not resolved to the species level.

#### 2.3.2 Diversity Analysis

Alpha and beta diversity indices were calculated at a rarefaction depth of 43,313 reads per sample using the QIIME2 plugin q2-diversity. A phylogenetic tree was constructed using the QIIME2 plugin q2-fragment-insertion (Matsen et al., 2010; Eddy, 2011; Matsen et al., 2012; Janssen et al., 2018) for phylogenetic alpha (Faith’s phylogenetic diversity) and beta diversity (UniFrac) measurements. Kruskal–Wallis rank-sum test was used to compare the differences between the alpha diversity indices among cohorts. The contribution of participant age, oral health status (healthy, OVH, and OSCC), and lifestyle factors (alcohol, betel nut, or cigarette consumption) were analyzed using Adonis with 9999 permutations. Distance-based permutational multivariate analysis of variance (PERMANOVA) was used to test the significant difference levels of the centroid of beta diversity metrics among cohorts in the ordination space of PCoA. For the observed significant PERMANOVA results, PERMDISP was then performed with 9999 permutations to determine the within-group homogeneity of dispersion. The Benjamini-Hochberg procedure was applied to control the FDR for multiple testing by statsmodels (0.10.2) (Seabold and Perktold, 2010). To evaluate the respective contribution of turnover and nestedness components to beta‐diversity as a whole, we calculated the multiple-site dissimilarity (Sørensen-based, β_SØR_), and the partitioning dissimilarities that accounted only for turnover (Simpson-based, β_SIM_) and for nestedness (β_NES_) components, respectively (Baselga, 2010).

#### 2.3.3 Core Microbiome Analysis

Core microbiome analysis was performed using a customized Python script and visualized using matplotlib-venn (0.11.5). The feature table was first converted to incidence data (presence/absence), and the prevalence of each taxon in each cohort was calculated. If the prevalence of a given taxon was greater than 75%, it was considered a core species in a cohort (Takeshita et al., 2016; Willis et al., 2018). A Venn diagram was used to illustrate the distinct and shared core species between cohorts.

#### 2.3.4 *In Silico* Metagenome Prediction

The metagenomic content was developed *in silico* from the denoised 16S rRNA genes using PICRUSt2 (Douglas et al., 2020). HMMER (www.hmmer.org), EPA-NG, and GAPPA were performed to place ASVs into reference phylogeny (Barbera et al., 2018; Czech and Stamatakis, 2019). The functional profiles of oral microbiome were predicted in accordance with the community-wide abundance method. The castor R package was subsequently used for hidden state prediction to infer gene family copy numbers (Louca and Doebeli, 2017). Finally, the EC number abundances were predicted based on the adjusted gene family abundances. To infer pathway abundances, MinPath was applied to identify a set of minimum pathways based on the predicted gene families (Ye and Doak, 2009). Default settings were used to regroup EC numbers to MetaCyc reactions and further inferred to MetaCyc pathway abundances (Caspi et al., 2020).

#### 2.3.5 Statistical Testing of Differential Abundance

LEfSe was applied to identify differentially abundant species and metabolic pathways among cohorts (Segata et al., 2011). The input of the frequency matrix was rarefied to the same depth and then transformed into a relative abundance matrix. The significance level was 0.05 for the Kruskal–Wallis test, and the cutoff of the logarithmic LDA scores was 3.

#### 2.3.6 Stochasticity Ratio Estimation

To evaluate the drivers of the community composition and functional profile, the null-model-based approach was used to calculate the normalized ratio of the difference between the actual and expected similarity, referring to as a selection strength (SS), to assess the strength of determinism acting against the stochastic forces (Ning et al., 2019). In this method, the actual Bray-Curtis similarity of any two samples in the metacommmunity of a cohort was first calculated based on taxonomic and pathway data and compared with the mean of null expected similarity that was obtained by averaging the similarity of 1,000 times of randomization of the two samples in the metacommunity. The stochasticity ratio was calculated as (1 - SS). The ratio ranges from 0 to 100%, with 0 for the community composition/functional profile solely shaped by deterministic processes, and 100% for the community composition/functional profile purely influenced by the stochastic forces. For the null model algorithm, proportional taxa/pathway occurrence frequency and richness were applied to generate random microbial/functional communities (Gotelli, 2000). Samples in each cohort shared the sample regional taxa/pathway pool in the null model algorithm.

### 2.4 Public Data Acquisition, Processing, and Re-Analysis

Academic search systems, including Google Scholar and PubMed, were used to find studies published in the last five years (2015–2020) with the search terms “oral microbiome”, “saliva microbiome”, and “OSCC”. We included 16S rRNA amplicon-based studies with publicly available sequences and metadata indicating OSCC or control for each sample. To compare the results, we only included studies with samples collected by non-invasive collection methods (oral swab, oral rinse, or saliva samples), while excluding the studies of using tissue biopsies and those without sample metadata. Studies with the OSCC cohort consisting of both the oral cavity and oropharynx types were also included. The raw sequence processing, diversity analysis, and core species/metabolic pathway analysis were performed as described in previous sections.

### 2.5 Prediction Using Machine Learning Analysis

The QIIME2 q2-sample-classifier plugin (Bokulich et al., 2018a) was used to predict sample health statuses based on taxonomic and functional profiles generated from this study, and the publicly available dataset was re-analyzed. Input data were randomly split into 80% for training and 20% for testing. The Random Forest classifier was applied for supervised machine learning. Cross-validated recursive feature elimination was applied for feature selection, with 5% of features eliminated at each iteration. Hyperparameters were automatically tuned using a random grid search with 5-fold cross-validation. Based on taxonomic and functional profiles, respectively, we performed the analysis procedure 100 times with different random seeds and recorded the testing accuracy ratio for each iteration. The resulting accuracy ratio data was tested using an independent *t*-test to determine the statistical significance of the machine learning prediction results. AUROC metrics were calculated using the scikit-learn package (Pedregosa et al., 2011). For multi-class classification, micro-average was used. The data in each study were trained and validated separately to minimize the experimental batch effects.

## 3 Results

### 3.1 Cohort Descriptions and Sequencing Quality

Saliva samples collected from 75 male participants, including healthy controls (normal, n = 27), non-recurrent OVH patients with > 8-year follow-up (2011 December–2019 November) (OVH, n = 21), and patients having primary OVH followed by OSCC development within eight years follow-up (OSCC, n = 27), were included in this study (**Table S1**). Statistical analysis of the participants’ metadata (age and lifestyle factors) showed that the differences in the studied cohorts were significant for age between normal and OSCC cohorts, and for betel nut chewing habits between OVH and OSCC cohorts (**Table S2**). Illumina high-throughput sequencing generated a total of 14,261,633 raw sequences targeting the V3–V4 region of the 16S rRNA gene. After sequence denoising, 8,522,211 denoised reads were retained from 75 samples, with an average of 113,629 ± 33,379 high-quality sequences per library. The plateau rarefaction curves indicated that the sequencing depth was sufficient for downstream analysis (**Figure S1**).

### 3.2 Phylogenetic Diversity Was Slightly Reduced in the Microbiomes of Diseased Cohorts

**Figure 1:**
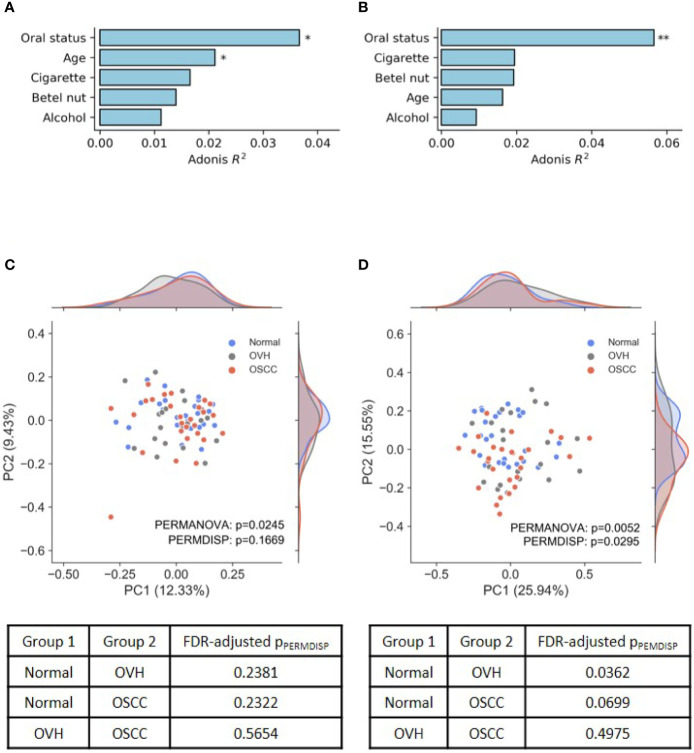
Differences in oral microbiomes among normal, OVH, and OSCC cohorts. **(A, B)** Adonis analysis based on **(A)** unweighted and **(B)** weighted UniFrac distance metrics shows the effect (*R^2^*) of factors with the oral microbiome. * indicates FDR-adjusted *p* < 0.05 and ** indicates FDR-adjusted *p* < 0.01. **(C, D)** Principal coordinate analysis (PCoA) plots of taxonomic profiles based on **(C)** unweighted and **(D)** weighted UniFrac distance metrics. Marginal kernel densities visualize the distribution of microbial diversity along both axes. The pairwise PERMDISP reveals the dispersion effect (FDR-adjusted *p* < 0.05) between normal and OVH cohorts.

In the assessment of ASVs detected within samples, the results revealed that the four alpha diversity indices were not significantly different between the studied cohorts (q > 0.05 after false discovery rate (FDR) adjustment, **Table S3**). To evaluate the effects of risk factors, including participant age, oral health status, and lifestyle, on the changes of the oral microbial community, UniFrac distance-based Adonis analysis was performed with the host health status as the last variables (Alcohol+BetelNut+Cigarette+Age+HealthStatus). As shown in **Figures 1A, B**, oral health status was detected as the strongest explanatory power (Adonis R^2^ = 0.037 for unweighted UniFrac and 0.057 for weighted UniFrac) to significantly differentiate the cohorts (FDR-adjusted *p*< 0.05). Although age and betel nut chewing habit exhibited significant distinction between some cohorts (**Table S2**), the variable, betel nut chewing, was not significantly associated with changes in oral microbial communities. However, age as a variable may confound the change of oral microbiome with the unweighted UniFrac distance (Adonis R^2^ = 0.021, FDR-adjusted *p*< 0.05). The UniFrac-based beta diversity distribution of salivary microbiomes from the cohorts was visualized using a principal co-ordinate analysis (PCoA) plot (**Figures 1C, D**), and showed random distribution on the ordination space. Pairwise permutation analyses of multivariate dispersions (PERMDISP) analysis further confirmed that the dispersion effect was not found among cohorts based on the unweighted UniFrac metric (p_PERMDISP_ = 0.1669); however, this effect was observed between normal and diseased (OVH/OSCC) cohorts. In particular, the dispersion effect reached a significant level between the normal and OVH cohorts (FDR-adjusted p_PERMDISP_ = 0.0362) in the weighted UniFrac distance measurement, suggesting heterogeneous dispersion of abundant taxa in salivary microbiota in correspondence with the oral health status.

### 3.3 Oral Carcinogenesis Altered Core Microbiomes

**Figure 2:**
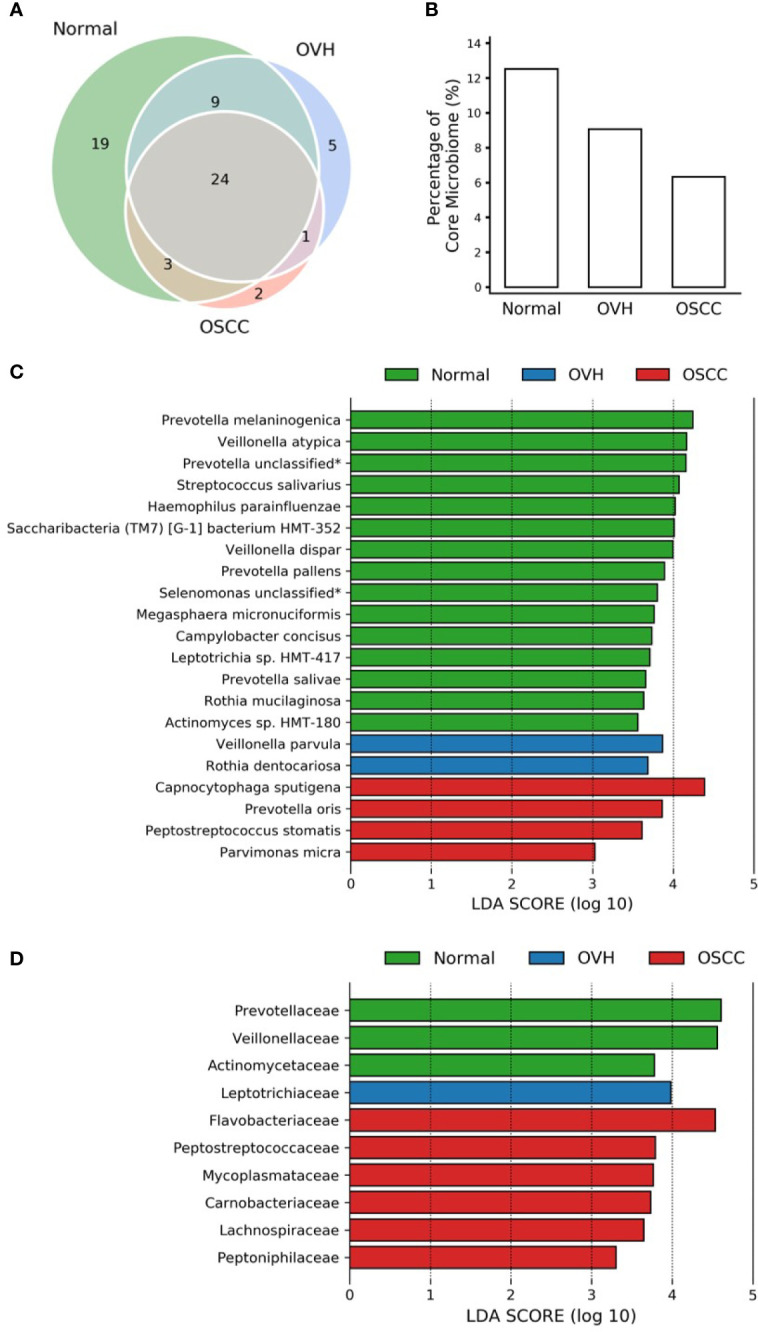
Core microbiome analysis. **(A)** Venn diagram of core microbiomes among cohorts. The core is defined as the species taxa present in saliva with ≥ 75% prevalence. **(B)** The fraction of core species number to overall species richness in each cohort. **(C)** LEfSe reveals the distribution of core species displaying the abundance significantly higher (LDA > log10^3^) among cohorts. The asterisk (*) indicates a taxon that was annotated only to the genus level. **(D)** Same as **(C)** but at the family level.

To further compare the differences in salivary microbiomes among cohorts, we investigated the “core” species, defined as the taxa commonly present in the saliva of each cohort with a prevalence > 75% (Takeshita et al., 2016; Willis et al., 2018). The number of core species was 55 (67.14 ± 11.06% of total abundance), 39 (47.24 ± 14.96% of total abundance), and 30 (44.52 ± 12.81% of total abundance) in normal, OVH, and OSCC cohorts, respectively, and 24 species taxa were universal in the saliva samples from all cohorts, even when the oral health status altered (**Figure 2A**). Five species (*Anaeroglobus geminatus*, *Porphyromonas gingivalis*, *Prevotella oulorum*, Saccharibacteria (TM7) [G5] bacterium HMT-356, and *Tannerella forsythia*) were specific to the OVH cohort. In comparison, two species (*Capnocytophaga sputigena* and *Catonella morbi*) were specific to the OSCC cohort. One species (*Dialister invisus*) was specific to the OVH and OSCC cohort. Interestingly, a decreased trend in overall core species richness (gamma diversity) was clearly observed with deteriorating oral health status (**Figure 2B**) from 12.53% in the normal cohort to 6.34% in the OSCC cohort.

The linear discriminant analysis effect size (LEfSe) analysis revealed the core species with significant abundance in each group (*p* < 0.05, LDA score > 10^3^) (**Figure 2C**). A total of 15 species taxa were enriched in the normal cohort compared to 2-4 species in the two diseased cohorts. In the normal cohort, they included two unclassified taxa related to *Prevotella* and *Selenomonas* genera, three uncultured species (*Saccharibacteria* (TM7) [G-1] bacterium HMT-352, *Leptotrichia* sp. HMT-417, and *Actinomyces* sp. HMT-180), and 10 known species within 7 genera: *Prevotella* (*Prevotella melaninogenica*, *Prevotella salivae*, and *Prevotella pallens*), *Veillonella (Veillonella atypica*, and *Veillonella dispar*), *Streptococcus salivarius*, *Haemophilus parainfluenzae*, *Megasphaera micronuciformis*, *Campylobacter concisus*, and *Rothia mucilaginosa*. For the OVH cohort, only *Veillonella parvula* and *Rothia dentocariosa* were significantly abundant (*p* < 0.05, LDA score > 10^3^), while four species, *Capnocytophaga sputigena*, *Prevotella oris*, *Peptostreptococcus stomatis*, and *Parvimonas micra* were specifically abundant in the OSCC cohort (abundance see **Table S4**). Furthermore, LEfSe conducted with higher rank data showed that the species variation converges at specific family-level taxa in different cohorts. When prevalence taken into account, the enriched family taxa in the normal cohort were *Actinomycetaceae* (mean ± SD; 1.55% ± 1.16%), *Veillonellaceae* (12.50% ± 7.41%), and *Prevotellaceae* (20.60% ± 10.00%). *Leptotrichiaceae* (3.05% ± 3.92%) was the only family specifically enriched in the OVH cohort. The saliva microbiome, however, shifted to *Flavobacteriaceae* (8.61% ± 11.36%), *Peptostreptococcaceae* (2.37% ± 1.61%), *Mycoplasmataceae* (1.46% ± 2.36%), *Carnobacteriaceae* (1.65% ± 2.12%), *Lachnospiraceae* (2.19% ± 1.83%), and *Peptoniphilaceae* (0.53% ± 0.51%) as abundant taxa specific to the OSCC cohort (**Figure 2D** and **Figure S2**).

### 3.4 Distinct Metabolic Pathways Were Dysregulated Among Three Cohorts

**Figure 3:**
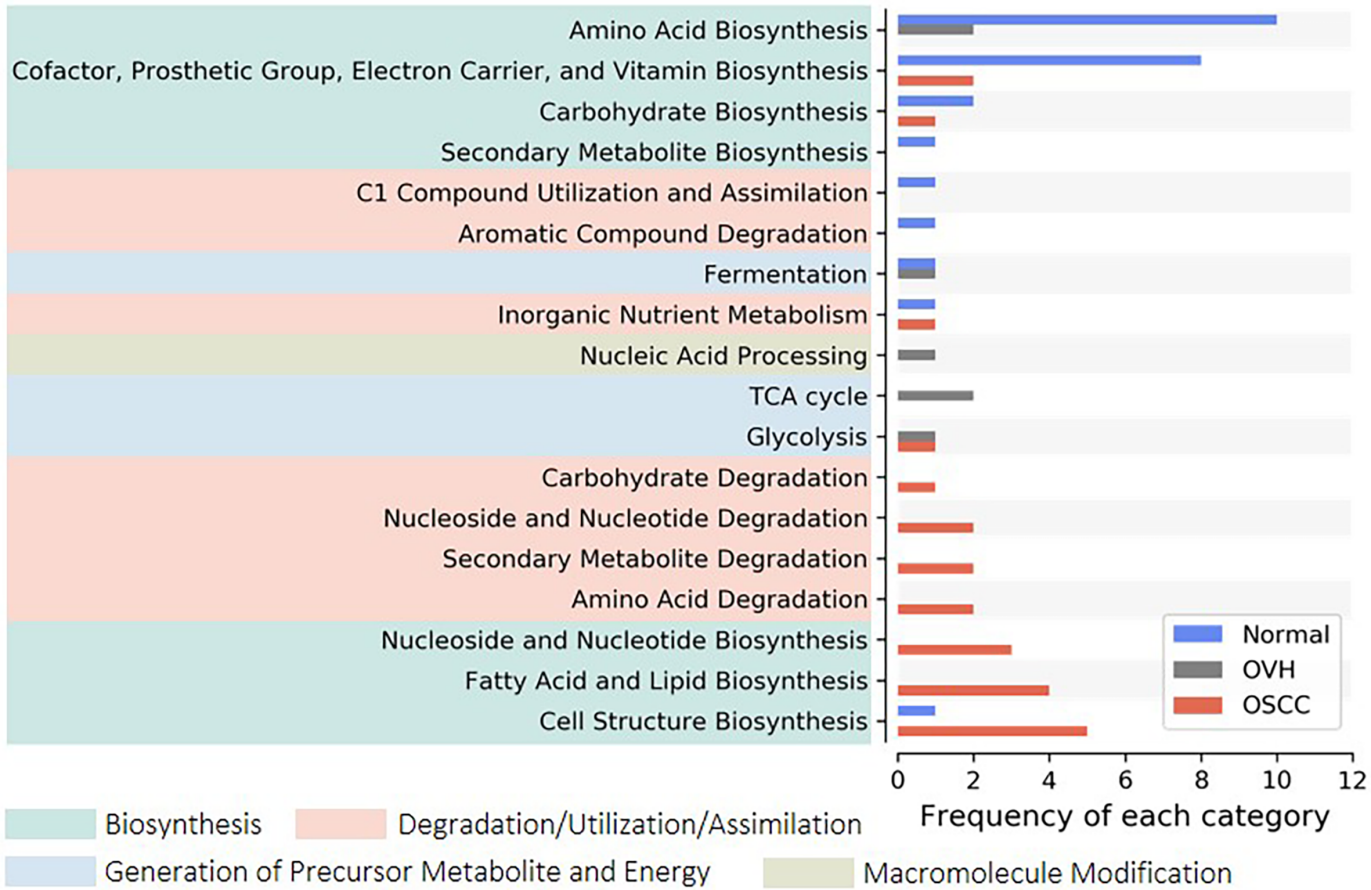
Distribution of signature pathways. The signature pathways, which abundances are significantly higher concerning each studied cohort, are detected using LEfSe. The inferred pathways are collapsed to each category based on Metacyc’s pathway ontology. Colored boxes indicate a higher rank of the categories.

We applied PICRUSt2, an updated version of a widely used metagenomic prediction tool (Langille et al., 2013), to infer the functional profiles of the microbial communities using denoised ASVs. The nearest-sequenced taxon index (NSTI) of 81.68% of reads was less than 0.15 (**Figure S3A**), suggesting the high-quality metagenome predicted (Langille et al., 2013). LEfSe analysis identified 26, 7, and 24 inferred pathways that were significantly abundant specific to normal, OVH, and OSCC cohorts, respectively (**Figure S3B**). By categorizing these pathways to higher classes, we found that most of them belong to amino acid biosynthesis (10 pathways), and cofactor, prosthetic group, electron carrier, and vitamin biosynthesis (8 pathways) for the normal cohort. The pathways for cell structure biosynthesis (5 pathways), fatty acid and lipid biosynthesis (4 pathways), and nucleoside and nucleotide metabolism (3 for biosynthesis; 2 for degradation) were abundant in the OSCC cohort (**Figure 3**). Only three pathways belonging to TCA cycles and nucleic acid processing were found to be significantly higher in abundance in specific relation to the OVH cohort.

### 3.5 Though a High Taxonomic Turnover, Functional Nestedness Evolved During Oral Carcinogenesis

**Figure 4:**
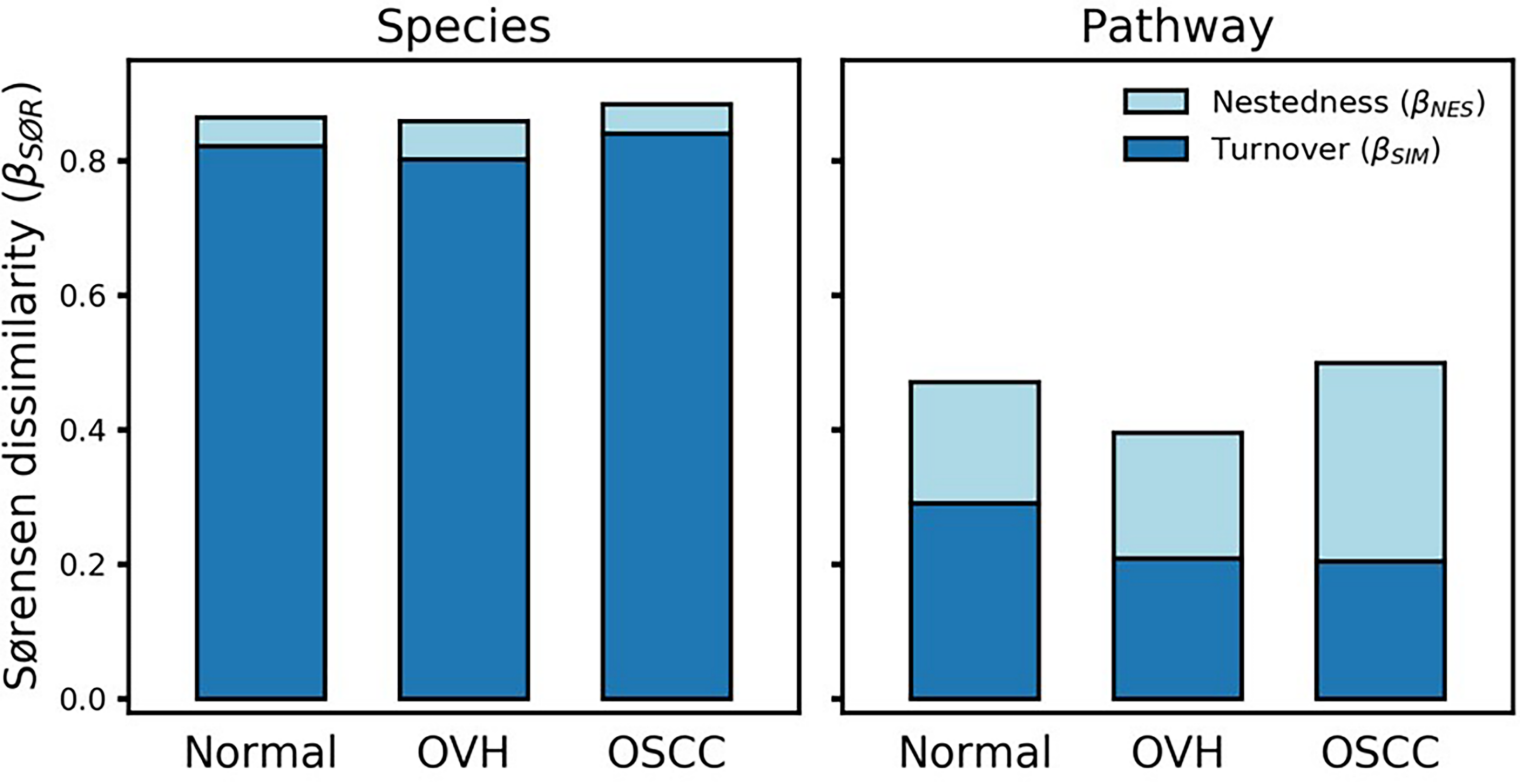
Multiple-site beta diversity (Sørensen dissimilarity) and corresponding nestedness and turnover components. The dissimilarities were analyzed in terms of species and metabolic pathway profiles in each cohort.

To determine the differentiation of beta diversity in the saliva microbiome, we compared the dissimilarities of the salivary microbiomes quantitatively based on taxonomic and functional profiles. For community composition data, the species nestedness was 0.042, 0.058, and 0.043 (sustainably lower than the turnover: 0.822, 0.802, and 0.841) for the normal, OVH, and OSCC cohorts, respectively (**Figure 4**), showing that the differentiation of salivary microbiomes was predominantly influenced by the species turnover. The high taxonomic turnover rate (low prevalence (< 33%) and high variation of species) among closely related species within these distinct family taxa could be visualized *via* a taxonomic tree (**Figure S4**). By contrast, the functional profiles of the salivary microbiomes were relatively stable: the mean multi-site Sørensen dissimilarity related to pathways (0.456 ± 0.044) was lower than that related to species taxa (0.869 ± 0.013) by approximately 50%. Notably, the numerical distributions of function nestedness (0.180, 0.187, and 0.295) and function turnover (0.291, 0.209, and 0.204) were relatively similar in each of the three cohorts (normal, OVH, and OSCC, respectively), as compared to species-based Sørensen dissimilarity (**Figure 4**). Conspicuously, the ratio of the function nestedness to turnover increased from 0.620 for normal or 0.896 for OVH, to 1.449 for OSCC, suggesting that nestedness emerges with the functional differentiation of salivary microbiomes during the process of oral carcinogenesis. Regardless of the observed high species turnover of the salivary ecosystem, the dominance of functional nestedness in the OSCC cohort suggests that a set of distinct microbial functions, which may be associated with oral carcinogenesis, evolved accordingly in the oral cavity.

### 3.6 Deterministic Processes Influence Functional Profiles but Not Taxonomic Variations

**Figure 5:**
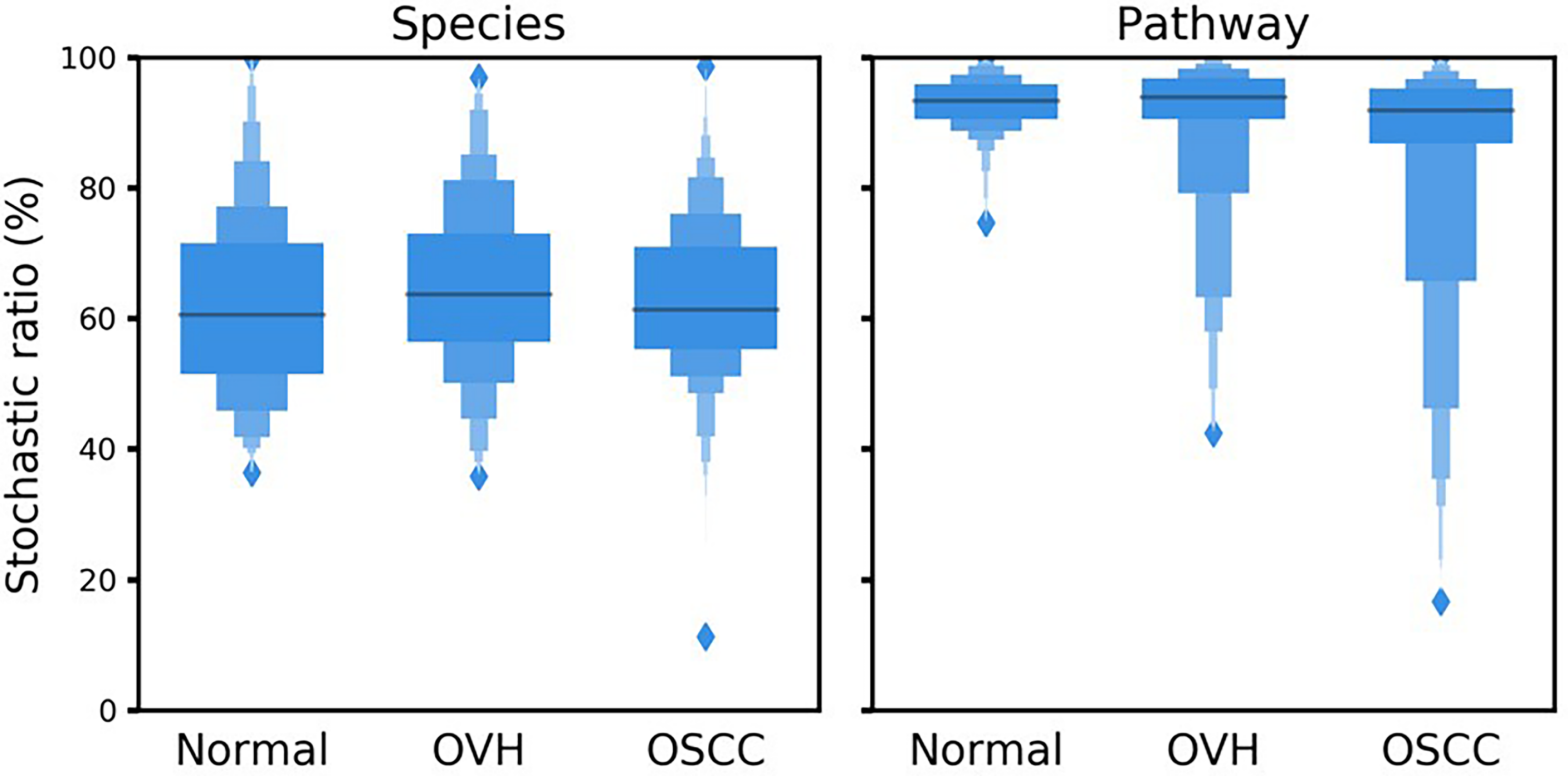
Boxplots illustrate the null-model-based stochastic ratio of microbial taxonomic composition and functional profile based on Bray–Curtis dissimilarities. The simulated procedure was repeated 999 times with proportional occurrence frequency and richness.

To better understand the driving forces in shaping the salivary microbiomes, a null model-based quantitative analysis of stochasticity with taxonomic and functional profiles was performed (Ning et al., 2019), respectively. The resulting stochasticity ratio serves as an index to assess the partition and contribution of the deterministic and stochastic processes in shaping the microbiome structure. In the quantitative assessment of the relative importance of the two ecological drivers, the deterministic and stochastic influence is summed to a total of 100%. Thus, the higher the stochasticity ratio, the stronger influence of stochastic processes or the less influence of deterministic forces. **Figure 5** shows that the stochastic process dominated the drivers of the bacterial community composition and functional gene content in saliva, with a stochastic ratio of inferred pathways (85.97 ± 16.73% to 93.04 ± 4.02%) higher than that of species taxa (61.88 ± 13.75 to 64.44 ± 13.11%). This finding suggests that the stochastic process played a more critical role in structuring the saliva microbiome at the functional level than the taxonomic level. Remarkably, the distribution patterns of taxa-based stochastic ratio among cohorts were relatively similar, showing stable stochastic and deterministic influences on taxonomic variations in the microbiomes of these three cohorts. By contrast, far broader distribution spectra for the function-based stochastic ratios were displayed in the strong association with the progression of oral carcinogenesis. Corresponding to the results of partitioning turnover and nestedness (**Figure 4**), this finding differentiates the stochastic influences from deterministic ones on shaping the saliva microbiome of different cohorts. It also supports a shift of the underlying driving force of the functional alternation towards the deterministic process corresponding to changes in oral health status from healthy through OPMD to OSCC.

### 3.7 Meta-Analysis Validates the Relation of Dysregulation Consistently to Several Taxa and Pathways

**Figure 6:**
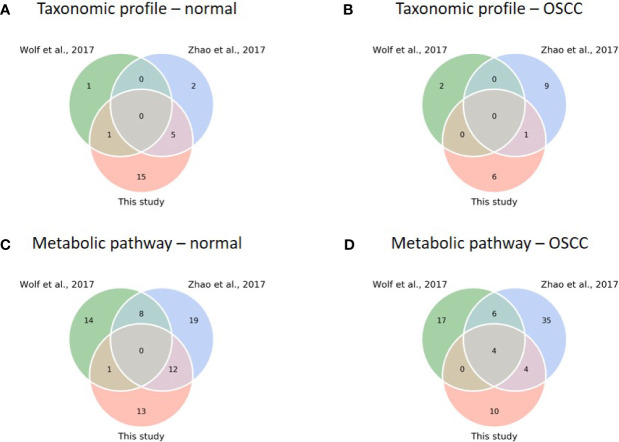
Comparison of diversity and core analysis between taxonomic and functional profiles from this study and previous studies (Wolf et al., 2017; Zhao et al., 2017). **(A–D)** Venn diagrams reveal common core species/pathways (prevalence > 75%) in normal and OSCC cohorts, respectively. (Taxonomic profiles of normal **(A)** and OSCC **(B)** cohorts; metabolic pathway profiles of normal **(C)** and OSCC **(D)** cohorts).

To test our findings’ generalizability, we compared the taxonomic and functional data of this study with two previous studies [(Wolf et al., 2017; Zhao et al., 2017); see justifications in **Table S5**]. Because all the previous studies focused on the analysis of 16S rRNA sequences with a 97% similarity threshold using a clustering approach to resolve the signature taxa at the genus level, we re-analyzed the sequences with our analytical pipeline to achieve phylogenetic resolution down to the species level. Although the signature species with specific abundances related to the study cohorts were found in each study, none was shared across all three studies for the normal and OSCC cohorts (**Figures 6A, B**), suggesting inconsistency among different studies. LEfSe was then conducted with family taxa. Similar to our data, re-analyzing Zhao’s data at the family level revealed that *Actinomycetaceae* (1.82% ± 1.79%) and *Veillonellaceae* (8.09% ± 6.89%) were enriched in the normal control, whereas *Flavobacteriaceae* (7.06% ± 7.01%), *Peptostreptococcaceae* (2.04% ± 1.80%), and *Peptoniphilaceae* (0.06% ± 0.95%) were abundant in the OSCC group. *Prevotellaceae* (28.39% ± 15.67%) and *Carnobacteriaceae* (1.29% ± 1.62%) were also enriched in their OSCC. The differential abundance of either family in normal versus OSCC cohorts between ours and Zhao’s data suggests that microbial turnover in saliva, albeit moderate, can still be detected even at the family level. For the re-work of Wolf’s data, the abundance of *Pasteurellaceae* and *Bifidobacteriaceae* was specific to normal and OSCC cohorts, respectively. The discrepancy between Wolf’s data and the other two studies (Zhao’s data and ours) could likely attribute to a low sample number and different cancer types (11 samples; oropharynx = 7, oral cavity = 4) studied.

Nevertheless, we found a comparatively high number of pathways between studies when comparing functional profiles. For the normal cohort, no common pathway across studies was detected (**Figure 6C**). For the OSCC cohort, four pathways involved in nucleotide biosynthesis (UMP biosynthesis I) and cell structure biosynthesis (UDP-N-acetylmuramoyl-pentapeptide biosynthesis I and II, and peptidoglycan biosynthesis I) were prevalent across the studies (**Figure 6D**). Importantly, we obtained consistent results in a functional meta-analysis (**Figure S5**). In Zhao’s and our datasets, amino biosynthesis pathways (arginine, ornithine, or histidine) were more abundant in normal control and pre-cancer groups, whereas the potentials of arginine and/or histidine degradation pathways were predicted higher in the OSCC cohort. In addition, two unsaturated fatty acid biosynthesis pathways (cis-vaccenate and gondoate) were abundant in the OSCC group, whereas no common pathway enriched in the cofactor or vitamin biosynthesis category was found. The meta-analysis results generalized our findings that microbiome dysbiosis in OSCC patients was dysregulated by the aforementioned distinctive functions. Because the PICRUSt2 predicted the functionality based on the abundance of the detected taxa, the functionality consequences are thus associated with the collective abundance of the contributing taxa. The family taxa, *Veillonellaceae* and *Actinomycetaceae* for the healthy cohort, and *Flavobacteriaceae* and *Peptostreptococcaceae* for the cancer cohort (**Figure S2**) might contribute to functional variations such as cofactor, prosthetic group, electron carrier, and vitamin biosynthesis, nucleoside and nucleotide biosynthesis, and cell structure biosynthesis (**Table S6**).

### 3.8 Using Functional Profiles Complements Using Taxonomic Data for Microbiome Analysis Among Different Studies

**Figure 7:**
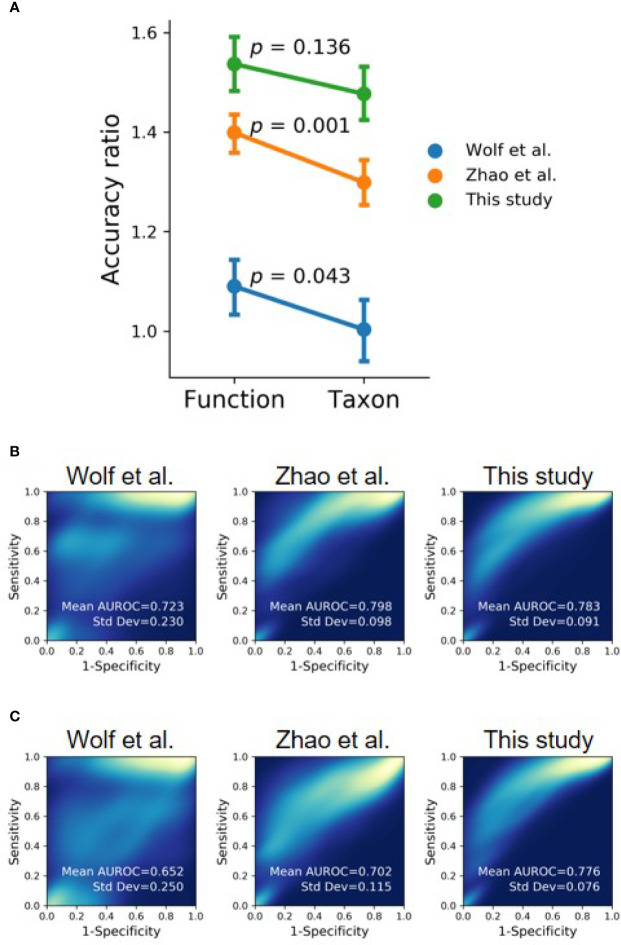
Evaluating functional profile as an alternative signature for OSCC detection using machine learning with the datasets of this study and previous studies (Wolf et al., 2017; Zhao et al., 2017). **(A)** The mean accuracy ratios of 100 iterations of the randomly split dataset (80% training and 20% testing) were based on taxonomic and functional profiles. The accuracy ratio is defined as the predicted accuracy to the accuracy of a random guess. Vertical bars indicate 95% confidence intervals. **(B, C)** The 2D-density plots of ROC curves from 100 iterations demonstrate a higher mean AUROC using **(B)** functional profiles to distinguish OSCC from normal cohorts compared to that using **(C)** taxonomic profiles.

To compare the performance of using taxonomic and functional profiles for predicting the occurrence of oral cancer, we trained random forest models with the profiles separately. The datasets from each study were independently processed in accordance with the analytical process illustrated in **Figure S6**. The mean accuracy ratio (the mean of the ratios of predicted accuracy to the accuracy of random guess, 100 iterations) was higher when using the functional profile than using the taxonomic profile (**Figure 7A** and **Figure S7**), although statistical significance was only detected in Wolf et al. (2017) (*t*-test, *p* = 0.043) and Zhao et al. (2017) (*t*-test, *p* = 0.001). Besides, we bootstrapped the receiver-operating characteristic (ROC) analysis 100 times. The area under the receiver operating characteristic curve (AUROC) was higher with functional profiles to distinguish OSCC samples from healthy controls, compared with that using taxonomic profiles (**Figures 7B, C**), suggesting the potential of using the (predicted) functional profiles can complement the use of taxonomic data in detecting the associations of the oral microbiome and health status.

## 4 Discussion

Previous studies have reported that the change of oral microbiome was associated with the influences mixed with oral diseases and risk factors (betel nut chewing, cigarette smoking, and alcohol consumption) (Sampaio-Maia et al., 2016; Wu et al., 2016; Hernandez et al., 2017; Fan et al., 2018; Debelius et al., 2020). In this study, however, by analyzing the saliva from 8-year follow-up cohorts, we found that the OVH carcinogenesis was identified as the main contributor to the altered oral microbiome. The age may be confounding, in particular for the changes in minor taxa in accordance with the significant level achieved by the Adonis analysis with unweighted UniFrac distance. Several studies have reported the association between age and salivary microbiome (Xu et al., 2015; Takeshita et al., 2016; Lira-Junior et al., 2018). This can partly be attributed to the functional decline of the immune system due to natural aging (Feres et al., 2016), and different levels of daily activities and metabolism between younger and elder people (Liu et al., 2020). Although the within-group diversity was not significantly different among cohorts, a decreased trend of core microbiome from healthy or OVH to OSCC was observed when the total diversity of each cohort (gamma diversity) was considered. Consistent with the previous studies that the richness of core species among healthy individuals was 9.6-13.1% (Takeshita et al., 2016; Willis et al., 2018) and it decreased to 5.96% in the OSCC patients with oral submucous fibrosis (Chen et al., 2020), our study also showed the decrease of richness from 12.53% in the normal cohort to 6.34% in the OSCC cohort. Together, the gamma diversity as an indication reflecting dysbiosis of the core salivary microbiome is effectually linked to the progression of oral cancer.

The altered oral microbiomes might be in part attributed to the host inflammation and immune responses to OPMD and OSCC. In periodontitis, the inflammatory environment was considered to be a source of host-derived nutrients for the microbes and thus altered the microbial community (Abusleme et al., 2013; Gaffen and Moutsopoulos, 2020). The change of micron-scale habitats in the oral cavity may subsequently alter the microbial composition and its related functional potentials. Common clinical features of OSCC including roughness and hardening of soft tissue, irregular ulcers, and exophytic tumors in the oral cavity (Bagan et al., 2010). The OVH was reported to form slightly elevated plaque-like lesions or protruding masses with the verrucous or papillary surface (Wang et al., 2009). The difference of the “landscape” in the oral cavity influences temperature, moisture, pH, oxygen, and nutrients availability and thus shapes the resident microbiota and, in turn, the neighboring microbes (Mark Welch et al., 2020; Wilbert et al., 2020).

Though we identified several enriched species that were associated with the health status of the oral cavity, the generalizability of these taxa as universal signatures for OSCC was suboptimal (**Figures 6A, B**). The signature taxa specifically related to the health status found in one study were not reported or even exhibited contradictory results to those in another study. For example, *P. melaninogenica*, *S. salivarius*, and *R.mucilaginosa* were highly abundant in patients with OPMD or OSCC (Mager et al., 2005; Pushalkar et al., 2012; Amer et al., 2020), but were identified as signatures for the healthy cohort in our dataset. Perera et al. reported that, at the species level, *Campylobacter concisus*, *Prevotella salivae*, *Prevotella loeschii*, and *Fusobacterium* oral taxon 204 were enriched in OSCC (Perera et al., 2018); however, the first two species were associated with healthy individuals in the present study. The genus *Actinomyces* was linked to tumor development in one study (Mukherjee et al., 2017), but the opposite microbial pattern was identified in another study (Zhao et al., 2017). The lack of consistency between studies could be attributed to the experimental design [e.g., sample types or hypervariable regions of the 16S rRNA gene (Tremblay et al., 2015)], the bioinformatics analysis pipeline [e.g., sequence denoising approaches (Nearing et al., 2018) and reference databases (Knight et al., 2018)], the genetics of studied cohorts [e.g., racial factors (Yang et al., 2019), and the complexity of oral carcinogenesis (Tanaka and Ishigamori, 2011)]. Alternatively, our results suggest that the inconsistencies may be due to the extremely high species turnover, which may be a consequence of the stochastic process (contributing to about 60%; **Figure 5**) in primarily shaping microbial communities in saliva (**Figure 4** and **Figure 5**). In addition to the host selection effect (contributing to about 40%; **Figure 5**), the high microbial species variation can be attributed to diet, lifestyles, hygiene habits, salivary dysfunction, frequent exposure to exogenous bacteria, and rapid changes in environmental factors (Marsh, 2003; Wade, 2013; Grassl et al., 2016; Lamont et al., 2018), eventually leading to high turnover rates of salivary microbiota, regardless of oral health status. Although the identified signature species were consistently related to several distinct family taxa, such as *Veillonellaceae* and *Actinomycetaceae*, in the healthy cohort; *Flavobacteriaceae*, *Peptostreptococcaceae*, and *Lachnospiraceae* in the OSCC cohort, the differential abundance of some signatures (e.g., *Prevotellaceae* and *Carnobacteriaceae*) showed opposite patterns in diseased versus normal cohorts when compared the previous studies with the meta-analysis in the present study (Zhang et al., 2020; Zhong et al., 2021). Taken together, microbes in saliva are subject to a high population dynamic at the taxonomic level, representing an extraordinarily dynamic ecosystem. Thus, it would be a challenging task to identify universal taxa signatures for OSCC.

The function profiles of saliva microbiomes remained distinguishable, despite the high taxonomic variation and abundance fluctuation in the saliva microbiome. This stable function profile but highly varied species composition in the salivary ecosystem may be likely due to functional redundancy, which has been proposed for other microbial ecosystems like soil and the human gut (Mendes et al., 2015; Vieira-Silva et al., 2016). Unlike the microbial composition, the functional profile is sensitive to the host oral carcinogenesis, as the proportion of driving forces shifted toward the deterministic process (**Figure 5**). This shift of drivers for the functional gene content may also reciprocally affect the primary contribution to the differences in functional profiles (**Figure 4**). Since nestedness reflects the effect of environmental filtering (Chase and Myers, 2011; Daniel et al., 2019), a possible explanation is that the diseased status of the oral cavity (OVH and OSCC) was a more influential environmental filtering factor than the healthy one, leading to the loss/gain of specific metabolic niches and an increase in nestedness components during the shift from healthy to diseased states. As a whole, oral carcinogenesis of OVH does not seem to impact the taxonomic composition but tilts the balance of functional gene content toward the deterministic process, making the functional profiles mirror oral cancer development more meritoriously than the taxonomic composition.

Through the re-analysis of publicly accessible OSCC-associated oral microbiomes datasets, we obtained more consistent results from the functional analysis. Although different hypervariable regions among studies may impact the analysis results to some extent, the advance in novel bioinformatics tool enables meta-analysis among multiple hypervariable regions (Callahan et al., 2017). The consistency can be explained by previous metabolomics studies (Lohavanichbutr et al., 2018; Song et al., 2020), which show dysregulated metabolite profiles in saliva between healthy and OSCC cohorts. Song et al. directly characterized the saliva metabolic profiles of healthy, precancerous, and OSCC cohorts, showing the specific down-regulated metabolites, including spermine, arginine, ornithine, and histidine, and the up-regulated metabolites, including putrescine, cadaverine, thymidine, adenosine, and 5-aminopentoate, in the OSCC cohort (Song et al., 2020). Several amino acid biosynthesis pathways (isoleucine, tryptophan, arginine, ornithine, valine, and methionine) were enriched in the normal group, particularly in Zhao’s and our datasets (**Figure S5**), suggesting potential up-regulation of those amino acids in the healthy cohort (and correspondingly down-regulation in the OSCC cohort), which is consistent with previous studies (Yang et al., 2018). Several previous studies also suggested the dysregulation of ornithine, arginine, and polyamine synthesis in the salivary microbiome during oral carcinogenesis from the healthy or precancerous stage (Chen et al., 2020; Sharma et al., 2020). In addition, Zhao’s and our study showed that the biosynthesis of lipids and fatty acids, especially cis-vaccenate and gondoate are enriched in the OSCC cohort (**Figure 3**). In the same line of the observations, the increase of cis-vaccenate would decrease the production of anti-inflammatory palmitoleic acid (Schirmer et al., 2016), leading to the increase of inflammatory cytokines like TNF-alpha required for the oral cancer stemness and aggressiveness (Lee et al., 2012; Krishnan et al., 2014). Although gondoate is often found in plants, it can also be anaerobically produced by microbes. Whether gondoate and the microbes responsible for the biosynthesis play any role in oral carcinogenesis remains characterized. The dysregulated metabolisms of amino acids, polyamines, long-chain fatty acids, and corresponding derivatives potentially underline the interplay among host oral carcinogenesis and oral microbiota and their metabolites. However, because the microbiome functions were analyzed with DNA samples through the PICRUSt2 prediction in this study, it is necessary to validate whether the corresponding pathways are expressed differentially in the salivary environments in accordance with the health status using paired DNA and RNA samples. Future studies would be warranted to address the dynamic interactions of the host, oral microbiome and metabolome, and how the microbial-host co-modulation of gene expressions and metabolites relate to the OSCC development.

In several previous studies, machine learning-aided models were trained for disease prediction using taxonomic profiles derived from 16S rRNA genes (Teng et al., 2015; Tremblay et al., 2015; Ai et al., 2017; Xu et al., 2018). Since the saliva microbiome was characterized as a highly dissimilar, high species-turnover, and stochasticity-dominated entity in this study, using taxonomic data as input features for classification and prediction tasks can be suboptimal and study-dependent. The predicted functional profiles from OSCC-associated individuals were reported similar despite the variation of the taxonomic profiles (Al-Hebshi et al., 2019). This study and a recent shotgun metagenomic study (Baker et al., 2021) suggest a high predictive accuracy for the health status using the functional profiles. These results suggest that the use of functional profiles may complement the use of taxa data to study the interplay of oral microbiome and OSCC. One limitation of using predicted functional profiles is the loss of function data attributed to a fraction of microbes without genomic information. This can be improved in the future by incorporating both metagenomics and culturomics to expand the microbiome database (Bilen et al., 2018). Raw sequence sharing, along with completely publicly available metadata, will also enable us to reproduce, compare, and validate results across studies through meta-analysis. This is especially crucial to untangle the roles of microbiomes in the progression from the healthy oral cavity through OPMD to OSCC.

Overall, this study has revealed the altered bacterial community composition with the specific functional dysregulation in saliva during OVH carcinogenesis. From the perspective of microbial ecology, any attempt to discover oral microbial consortia as biomarkers for oral cancer would be a daunting task due to the high taxonomic turnover (i.e., high variance and fluctuation) of the oral ecosystems. Functional gene content is relatively stable but susceptible to oral carcinogenesis, thus making functional profiles, although obtained by a prediction analysis in this study, a complement to taxa data in reflecting the oral cancer development. The dysregulated pathways identified in this study provided clues to study the interplay of the oral microbiome, metabolites, and oral cancer in the future.

## Data Availability Statement

The datasets presented in this study can be found in online repositories (https://github.com/mft-lab/OVH-study). The names of the repository/repositories and accession number(s) can be found below: https://www.ebi.ac.uk/ena, PRJEB39064.

## Ethics Statement

The studies involving human participants were reviewed and approved by Institutional Review Board of Chi Mei Medical Center (IRB No.: 10612-L02). The patients/participants provided their written informed consent to participate in this study.

## Author Contributions

J-HW, Y-LC, and L-WW conceived the study. J-HW designed the analysis. W-FC collected and processed the saliva samples. J-WC performed experiments and data analysis. J-WC and J-HW drafted the manuscript. J-HW and L-WW revised the manuscript and edited the final version. All authors contributed to the article and approved the submitted version.

## Funding

This study was supported by the Ministry of Science and Technology, Taiwan (grant numbers 107-2321-B-006-007, 108-2321-B-006-010, and 108-2221-E-006 -160 -MY3). The research was supported in part by Higher Education Sprout Project, Ministry of Education to the Headquarters of University Advancement at National Cheng Kung University (NCKU).

## Conflict of Interest

The authors declare that the research was conducted in the absence of any commercial or financial relationships that could be construed as a potential conflict of interest.

## Publisher’s Note

All claims expressed in this article are solely those of the authors and do not necessarily represent those of their affiliated organizations, or those of the publisher, the editors and the reviewers. Any product that may be evaluated in this article, or claim that may be made by its manufacturer, is not guaranteed or endorsed by the publisher.
