# Supplementary material for: Taxonomic and Functional Dysregulation in Salivary Microbiomes During Oral Carcinogenesis
Source: Front Cell Infect Microbiol. 2021 Sep 16;11:663068. doi: 10.3389/fcimb.2021.663068 (PMC8482814; doi:10.3389/fcimb.2021.663068)
Supplement: Supplementary file 1 [file Image_1.pdf]

Supplementary Figure S1

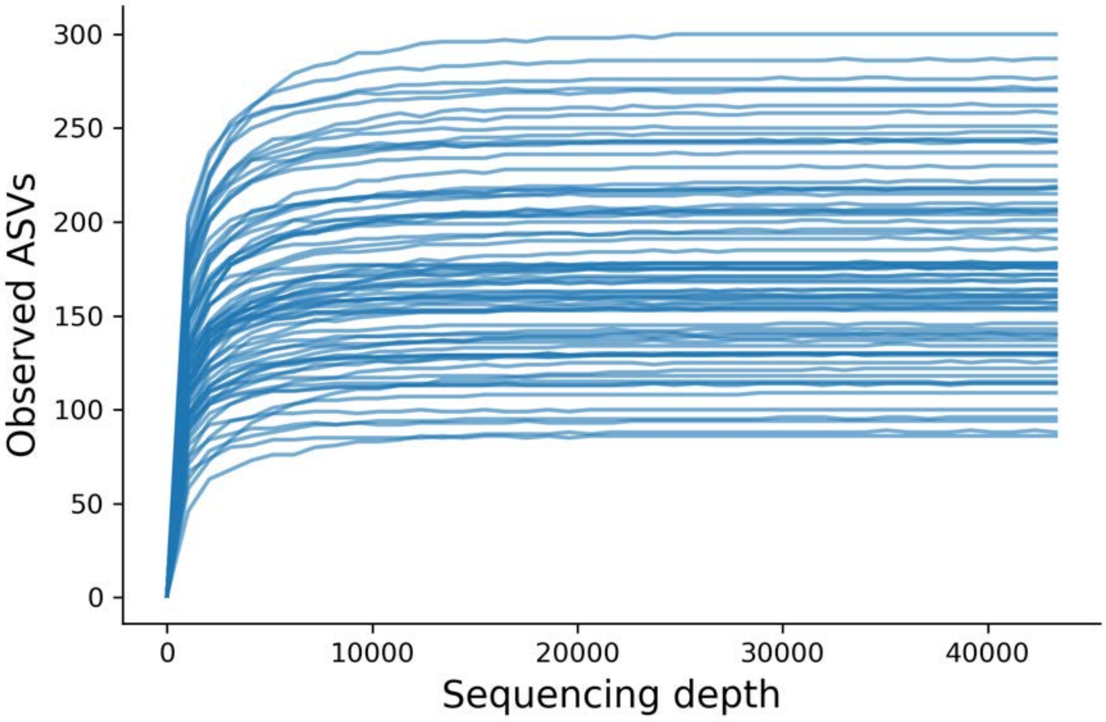

Figure S1. Alpha diversity rarefaction curves for 75 samples. The plateau curves indicate that sequencing depth was sufficient.

Supplementary Figure S2

A

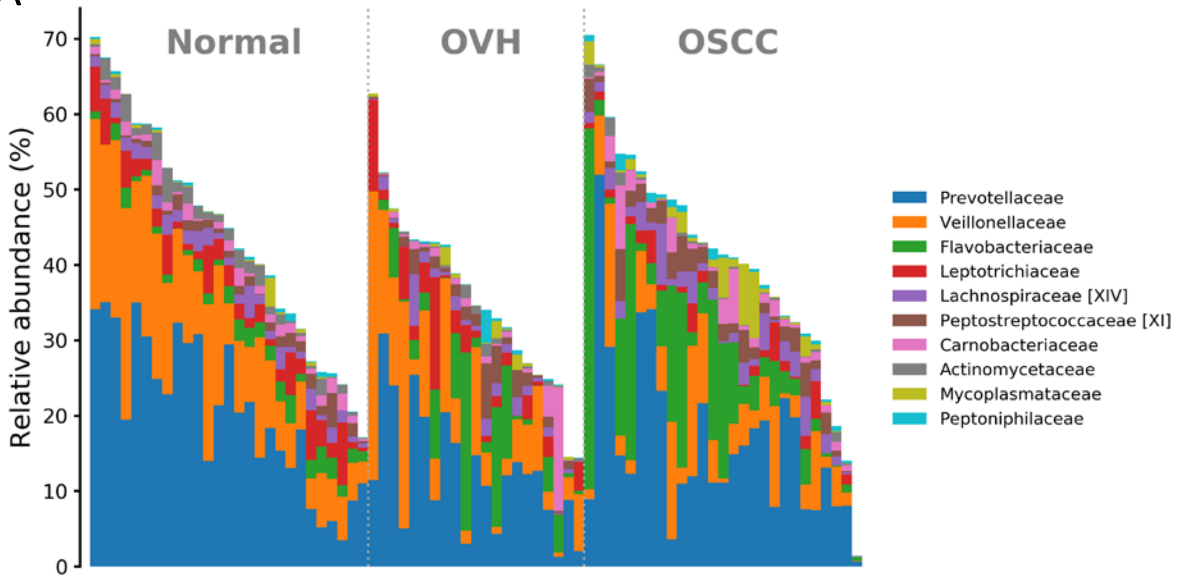

B

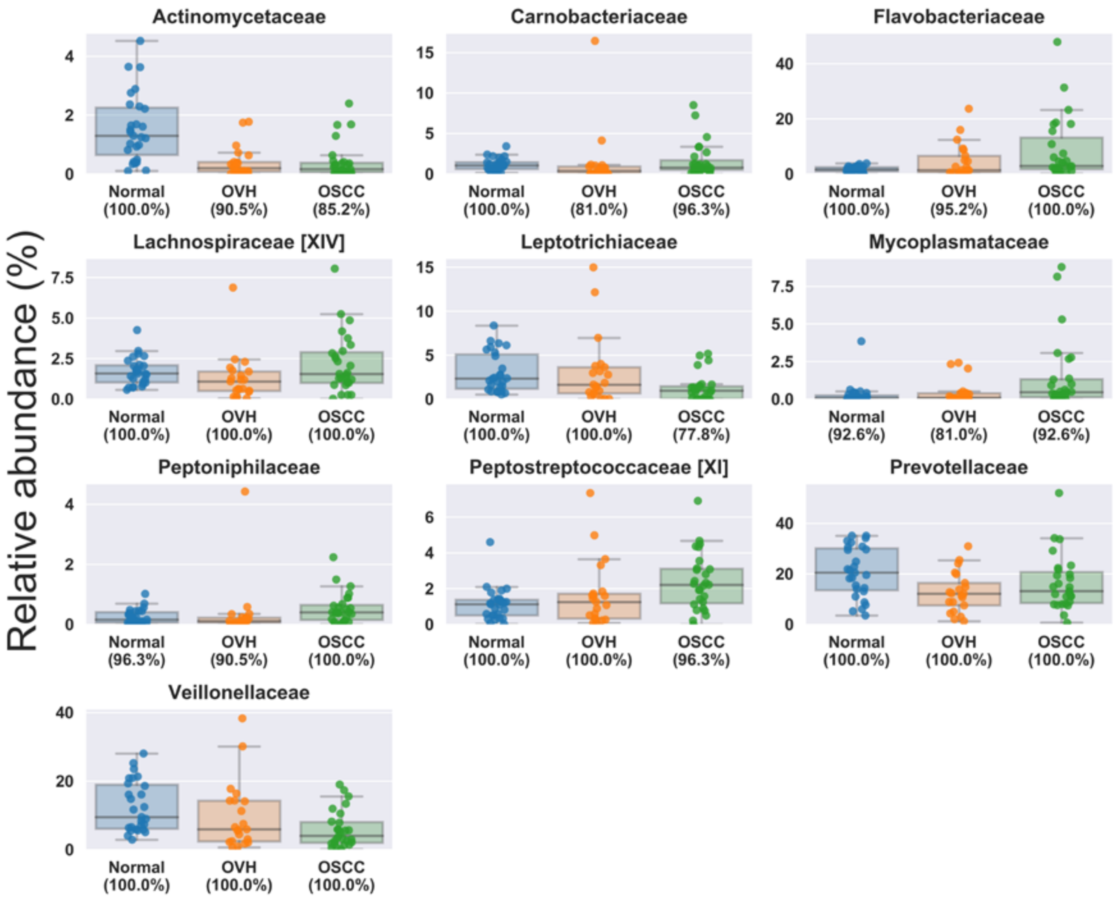

Figure S2. The variation of core families revealed by LEfSe. (A) Stacked bar plots show the relative abundance of core families. (B) Box plots with superimposed dot plots showing the relative abundance of each family among cohorts. The boxes represent the interquartile range (IQR), the horizontal line inside the box defines the median, and whiskers indicate the minimum and maximum values within 1.5 × IQR from both ends of the box. Each dot represent a sample. The prevalence of a family in given cohort is denoted as percentage in parentheses.

Supplementary Figure S3

A

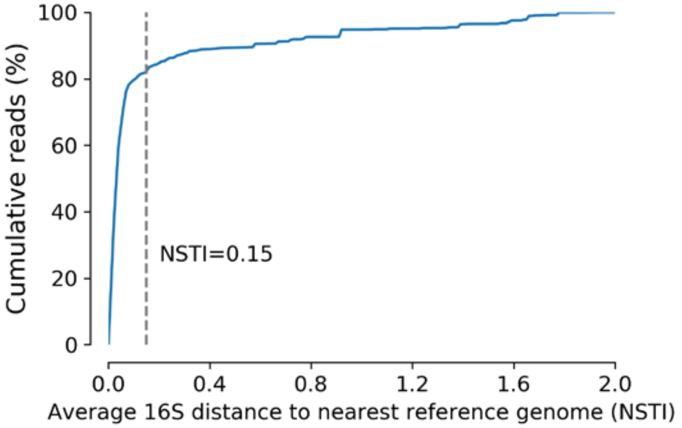

B

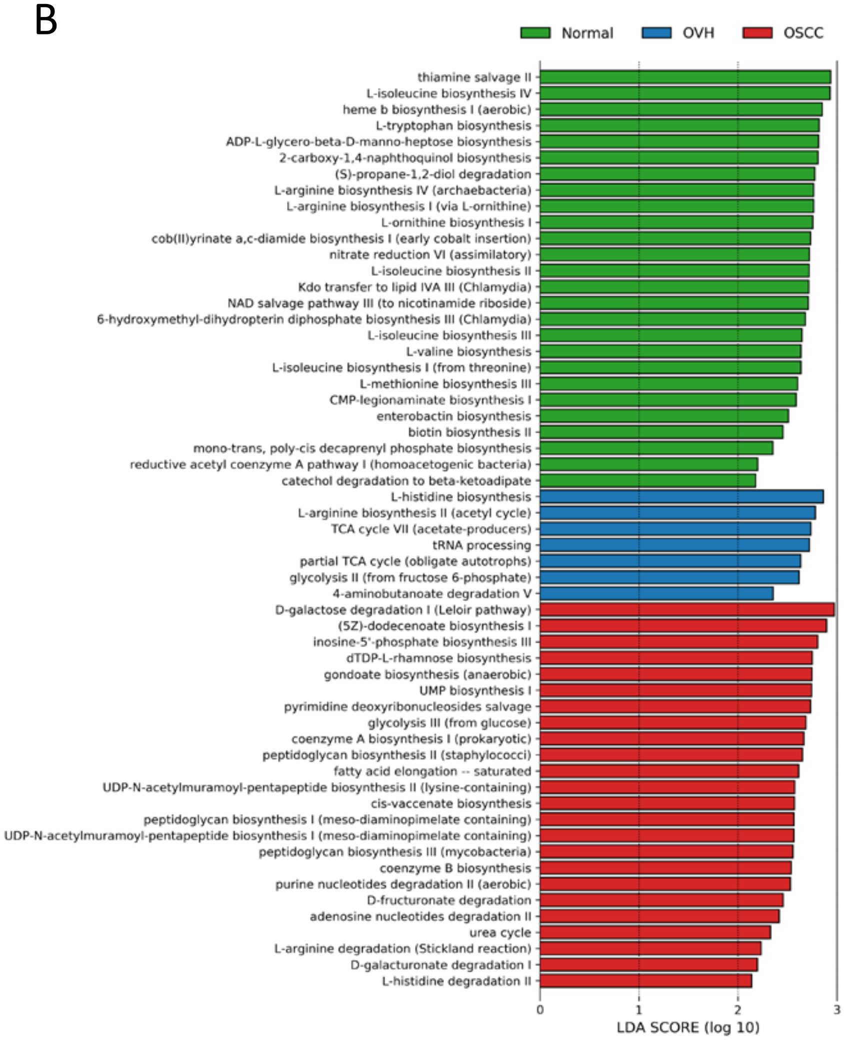

Figure S3. Prediction of metagenome using PICRUSt2. **(A)** The cumulative relative reads count shows 81.68% of reads with NSTI < 0.15, which indicates high to moderate quality of predicted metagenome. **(B)** LEfSe revealed significant metabolic pathways among cohorts.

Supplementary Figure S4

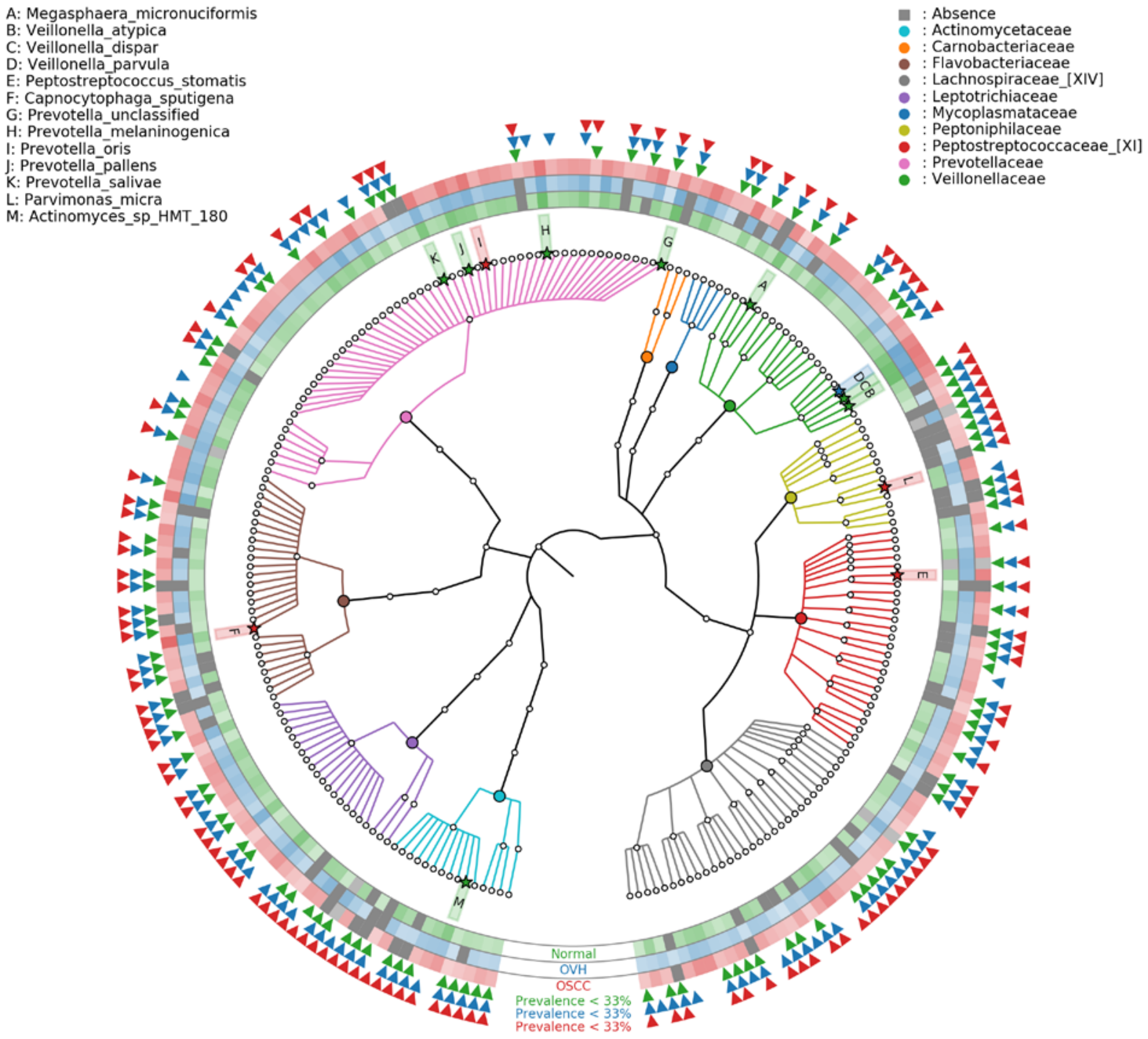

Figure S4. The taxonomic tree of core families revealed by LEfSe. The colored nodes and edges indicate the family of taxa in the clade. The stars indicate taxa with significant differential abundance revealed by LEfSe. The outer ring colors indicate cohorts (green, Normal; blue, OVH; red, OSCC). The lightness of ring color represents the relative abundance the the taxon (see color key). The outer triangles highlight the low prevalent taxa (< 33%) in the cohort.

Figure S5

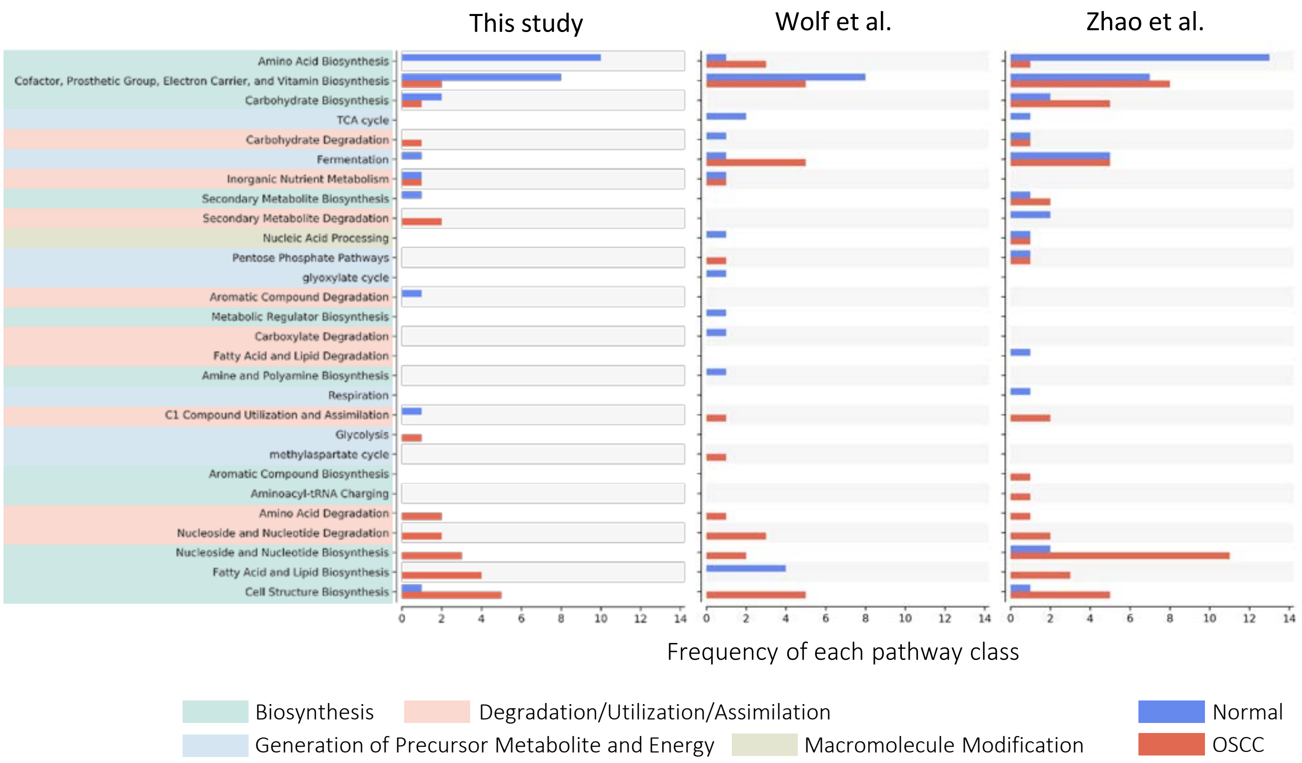

Figure S5. Meta-analysis reveals similar signature pathways. The signature pathways are detected using LEfSe as previously described. The inferred pathways are collapsed to each category based on Metacyc's pathway ontology. Colored boxes indicate a higher rank of the categories. The pathway counts in the first column (This study) is the same as that in Figure 3.

Supplementary Figure S6

A

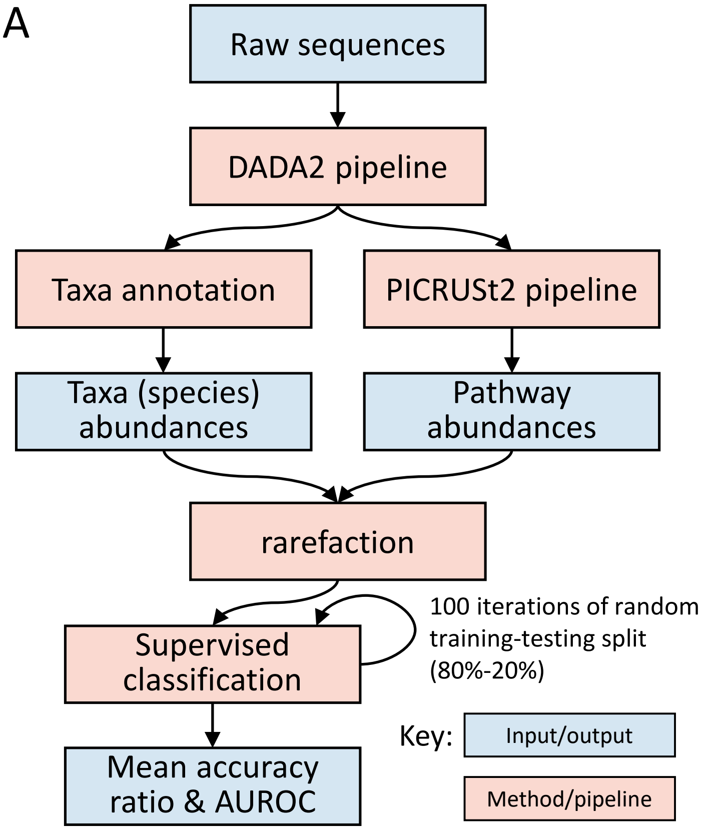

Figure S6. Flow chart illustrating the process of obtaining predicted results from published sequences. Blue boxes indicate input or output files, and red boxes indicate bioinformatics methods or pipelines.

Supplementary Figure S7

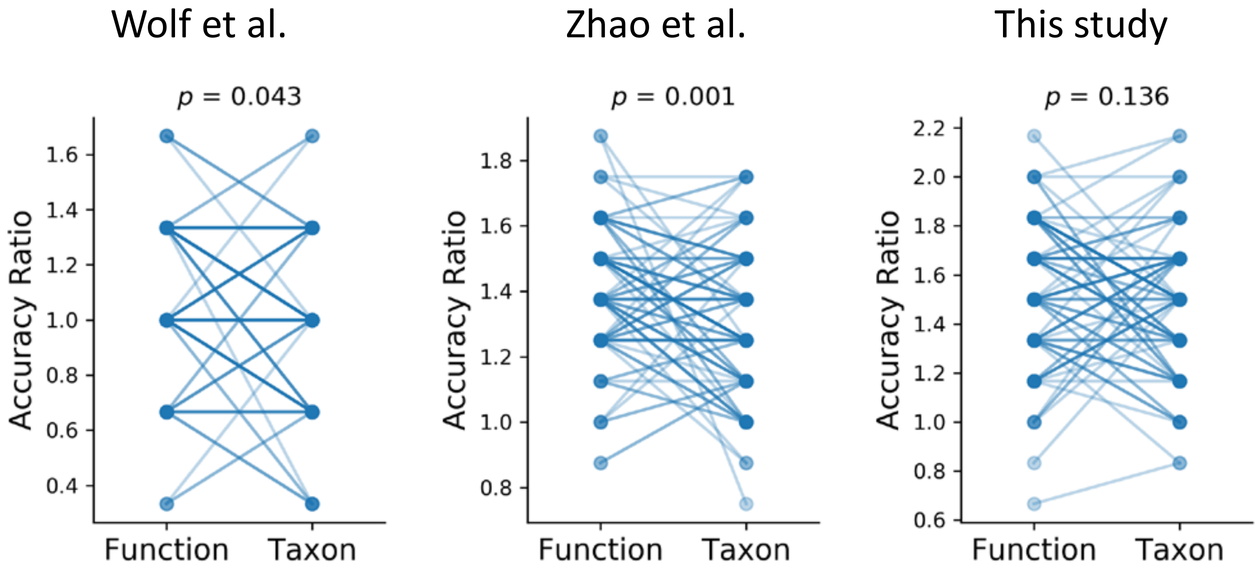

Figure S7. Pairwise comparisons of accuracy ratio between taxonomic and functional profiles. A total of 100 iterations was repeated with different training-testing splits (80%-20%) for each study. Independent t-test was performed to examined the statistical significance.
