# Supplementary material for: Taxonomic and Functional Dysregulation in Salivary Microbiomes During Oral Carcinogenesis
Source: Front Cell Infect Microbiol. 2021 Sep 16;11:663068. doi: 10.3389/fcimb.2021.663068 (PMC8482814; doi:10.3389/fcimb.2021.663068)
Supplement: Supplementary file 2 [file Table_1.pdf]

## Supplementary Materials for Manuscript Entitled

# Taxonomic and functional dysregulation in salivary microbiomes during oral carcinogenesis

Jiung-Wen Chen[a], Jer-Horng Wu[a]#, Wei-Fan Chiang[bf], Yuh-Ling Chen[c], Wei-Sheng Wu[d], Li-Wha Wu[e]#

[a] Department of Environmental Engineering, National Cheng Kung University, Tainan, Taiwan

[b] Department of Oral & Maxillofacial Surgery, Chi-Mei Medical Center, Liouying, Taiwan

[c] Institute of Oral Medicine, College of Medicine, National Cheng Kung University, Tainan, Taiwan

[d] Department of Electrical Engineering, National Cheng Kung University, Tainan, Taiwan

[e] Institute of Molecular Medicine, College of Medicine, National Cheng Kung University, Tainan, Taiwan

[f] School of Dentistry, National Yang-Ming University, Taipei, Taiwan

Running Head: Oral Microbiome in Malignant Transformation of OVH

#Address correspondence to Jer-Horng Wu (enewujh@ncku.edu.tw) or Li-Wha Wu (liwhawu@mail.ncku.edu.tw)

Postal address: No.1, University Road, East District, Tainan City 701, Taiwan

Table S1. Descriptive data of the study cohort. The former drinker/smoker/betel nut chewer indicates those who had quit drinking, smoking, or betel nut at the time of interview. The current one indicates those who still have the habits occasionally or regularly.

| Cohort | Sex | Age | Alcohol | Betel nut | Cigarette | Cancer stage |
|---|---|---|---|---|---|---|
| Normal (n=27) | Male=27 | 47.4±9.6 | never=14<br>former=1<br>current=12 | never=9<br>former=4<br>current=14 | never=8<br>former=4<br>current=15 | N/A |
| OVH (n=21) | Male=21 | 51.0±13.2 | never=6<br>former=1<br>current=14 | never=1<br>former=5<br>current=15 | never=2<br>former=2<br>current=17 | N/A |
| OSCC (n=27) | Male=27 | 54.4±10.1 | never=12<br>former =3<br>current=12 | never=3<br>former=21<br>current=3 | never=3<br>former=8<br>current=16 | I = 12<br>II = 6<br>III = 2<br>IV= 7 |
| Overall (n=75) | Male=75 | 50.9±11.2 | never=32<br>former=5<br>current=38 | never=13<br>former=30<br>current=32 | never=13<br>former=14<br>current=48 | I = 12<br>II = 6<br>III = 2<br>IV= 7 |

* N/A: not applicable

Table S2. Pairwise comparison of cohort metadata using the univariate analysis.

| Group 1 | Group 2 | Age | | Alcohol | | Betel nut | | Cigarette | |
|---|---|---|---|---|---|---|---|---|---|
| | | t-statistic | p-value | H-statistic | p-value | H-statistic | p-value | H-statistic | p-value |
| Normal (n=27) | OVH (n=21) | 1.117 | 0.270 | 2.521 | 0.112 | 3.265 | 0.071 | 3.6 | 0.058 |
| Normal (n=27) | OSCC (n=27) | 2.624 | 0.011 | 0.084 | 0.772 | 1.254 | 0.263 | 0.59 | 0.442 |
| OVH (n=21) | OSCC (n=27) | 0.996 | 0.325 | 1.973 | 0.16 | 15.468 | 8.39E-05 | 2.073 | 0.150 |

Table S3. Pairwise comparison of alpha diversity indices using the Kruskal-Wallis test.

| Group 1 | Group 2 | Pielou's evenness | | | Observed ASVs | | | Shannon's index | | | Faith's phylogenetic diversity | | |
|---|---|---|---|---|---|---|---|---|---|---|---|---|---|
| | | H | p-value | q-value | H | p-value | q-value | H | p-value | q-value | H | p-value | q-value |
| Normal (n=27) | OVH (n=21) | 0.0390 | 0.843 | 0.843 | 4.065 | 0.044 | 0.131 | 1.058 | 0.304 | 0.455 | 1.190 | 0.275 | 0.485 |
| Normal (n=27) | OSCC (n=27) | 2.0867 | 0.149 | 0.446 | 2.425 | 0.119 | 0.179 | 3.144 | 0.076 | 0.229 | 0.081 | 0.775 | 0.775 |
| OVH (n=21) | OSCC (n=27) | 0.7085 | 0.400 | 0.600 | 0.691 | 0.406 | 0.406 | 0.048 | 0.827 | 0.827 | 0.975 | 0.324 | 0.485 |

q-value is the FDR-adjusted p-value for multiple comparisons.

Table S4. Signature species with relative abundance (Mean ± SD, %) in the studied cohorts.

| Species | Normal | OVH | OSCC |
|---|---|---|---|
| *Parvimonas micra* | 0.13±0.13 | 0.26±0.75 | 0.33±0.31 |
| *Actinomyces* sp. HMT-180 | 0.86±0.76 | 0.10±0.16 | 0.18±0.40 |
| *Peptostreptococcus stomatis* | 0.14±0.19 | 0.31±0.86 | 0.90±1.15 |
| *Rothia mucilaginosa* | 1.40±1.87 | 0.52±0.76 | 0.63±1.62 |
| *Prevotella salivae* | 1.70±1.42 | 0.65±0.73 | 1.34±1.78 |
| *Rothia dentocariosa* | 0.97±1.85 | 1.47±2.53 | 0.48±0.95 |
| *Leptotrichia* sp. HMT-417 | 1.18±1.85 | 0.45±0.73 | 0.25±0.55 |
| *Campylobacter concisus* | 1.79±1.27 | 0.60±0.86 | 0.75±1.04 |
| *Megasphaera micronuciformis* | 1.68±1.70 | 0.48±0.56 | 0.51±0.81 |
| *Selenomonas* unclassified* | 1.50±1.30 | 1.49±1.87 | 0.59±0.46 |
| *Prevotella oris* | 0.57±0.62 | 0.91±0.83 | 1.98±2.27 |
| *Veillonella parvula* | 1.35±1.56 | 2.41±3.67 | 0.82±1.96 |
| *Prevotella pallens* | 1.90±3.60 | 0.61±1.08 | 1.52±3.92 |
| *Veillonella dispar* | 3.62±3.33 | 1.76±2.12 | 1.67±2.56 |
| *Saccharibacteria* (TM7) [G-1] bacterium HMT-352 | 2.98±3.57 | 0.99±2.35 | 0.89±2.63 |
| *Haemophilus parainfluenzae* | 2.85±3.16 | 1.15±2.37 | 1.51±2.34 |
| *Streptococcus salivarius* | 2.40±4.58 | 0.88±1.76 | 0.22±0.36 |
| *Prevotella* unclassified* | 3.49±4.60 | 1.54±2.74 | 0.63±0.79 |
| *Veillonella atypica* | 3.99±4.13 | 3.04±5.75 | 1.15±2.10 |
| *Prevotella melaninogenica* | 4.75±5.61 | 1.40±1.89 | 2.20±3.70 |
| *Capnocytophaga sputigena* | 0.24±0.31 | 0.70±1.15 | 4.69±9.49 |

The asterisk (*) indicates a taxon that was annotated only to the genus level.

Table S5. Studies related to the oral microbiome and oral cancer since 2015. Only studies with samples collected by non-invasive collection methods (oral swab, oral rinse, or saliva samples) was used in the present study. Data sets were downloaded on April, 2020

| Ref. | Year | Sample type | Platform | HVR | Accession number | Comment | Used in this study |
|---|---|---|---|---|---|---|---|
| [1] | 2015 | tissue | Roche 454 | V1-V3 | SRA204252 | Not Illumina platform, tissue sample | No |
| [2] | 2016 | tissue and saliva | Roche 454 | V3-V5 | NA | - | No |
| [3] | 2016 | saliva | Illumina MiSeq | NA | NA | - | No |
| [4] | 2017 | saliva | Illumina TruSeq | V4 | SRP107079 | Merged sequences, not suitable for DADA2 pipeline | No |
| [5] | 2017 | saliva and swab | Roche 454 | V3-V5 | SAMN06127413 | Not Illumina platform, no sample barcodes | No |
| [6] | 2017 | oral rinse | Illumina MiSeq | V4 | PRJNA321193 | Dubious sequences | No |
| [7] | 2017 | saliva | Illumina MiSeq | V4 | PRJEB18476 | Illumina platform, saliva samples | Yes |
| [8] | 2017 | swab | Illumina MiSeq | V4-V5 | SRP097643 | Illumina platform, oral swab samples | Yes |
| [9] | 2017 | tissue | Illumina MiSeq | V1-V3 | PRJNA352375 | Tissue sample | No |
| [10] | 2017 | swab | Illumina MiSeq | V1-V2 | PRJNA394711 | Healthy and precancer cohort, no cancer cohort | No |
| [11] | 2017 | saliva | Roche 454 | V3-V5 | NA | - | No |
| [12] | 2017 | tissue | Ion Torrent | V4 | Upon request | Not Illumina platform, tissue sample | No |
| [13] | 2017 | tissue | Clone | V1-V4 | LT674587-LT699699 | Not Illumina platform, tissue sample | No |
| [14] | 2018 | oral rinse | Illumina MiSeq | V6-V8 | NA | - | No |
| [15] | 2018 | tissue | Illumina MiSeq | V1-V3 | PRJNA415963 | tissue sample | No |
| [16] | 2018 | oral rinse | Illumina MiSeq | V3-V4 | NA | - | No |
| [17] | 2018 | saliva | Illumina MiSeq | V4 | NA | - | No |
| [18] | 2018 | oral rinse | Roche 454 | V3-V4 | NA | - | No |

| | | | | | | | |
|---|---|---|---|---|---|---|---|
| [19] | 2018 | saliva | Illumina MiSeq | V3-V5 | NA | - | No |
| [20] | 2019 | saliva | Illumina MiSeq | V1-V2 | NA | - | No |
| [21] | 2019 | saliva | Illumina MiSeq | V4 | NA | - | No |
| [22] | 2019 | oral rinse | Roche 454 | V3-V4 | NA | - | No |
| [23] | 2020 | swab | Illumina MiSeq | V3-V4 | PRJNA533177 | No data found | No |

Table S6 Multiple linear regression of the functionality and family taxa detected in the oral microbiomes.

| | Cofactor, Prosthetic Group, Electron Carrier, and Vitamin Biosynthesis | | |
|---|---|---|---|
| Coefficients | Normal | NRVH | OSCC |
| X0 (constant) | 14.846 | 13.9536 | 11.2937 |
| X1 (Actinomycetaceae) | **-0.1425** | **-1.1287** | **-0.7065** |
| X2 (Flavobacteriaceae) | -0.0556 | 0.0732 | 0.0342 |
| X3 (Peptostreptococcaceae) | -0.0301 | -0.006 | **0.442** |
| X4 (Veillonellaceae) | 0.0119 | 0.0148 | **0.1761** |
| R-squared | 0.047 | 0.259 | 0.339 |
| adjusted R-squared | -0.126 | 0.074 | 0.219 |

| | Nucleoside and Nucleotide Biosynthesis | | |
|---|---|---|---|
| Coefficients | Normal | NRVH | OSCC |
| X0 (constant) | 13.793 | 13.1739 | 16.4389 |
| X1 (Actinomycetaceae) | **0.1153** | **0.3727** | **1.0284** |
| X2 (Flavobacteriaceae) | **0.125** | -0.0116 | -0.041 |
| X3 (Peptostreptococcaceae) | **-0.3399** | **0.3314** | **-0.4029** |
| X4 (Veillonellaceae) | -0.0053 | 0.0037 | **-0.1562** |
| R-squared | 0.271 | 0.343 | 0.389 |
| adjusted R-squared | 0.138 | 0.179 | 0.278 |

| | Cell Structure Biosynthesis | | |
|---|---|---|---|
| Coefficients | Normal | NRVH | OSCC |
| X0 (constant) | 6.7241 | 6.6351 | 7.9722 |
| X1 (Actinomycetaceae) | 0.0549 | **0.4687** | **0.4458** |
| X2 (Flavobacteriaceae) | 0.0388 | -0.0242 | -0.0304 |
| X3 (Peptostreptococcaceae) | **-0.1245** | 0.037 | **-0.1709** |
| X4 (Veillonellaceae) | 0.0194 | 0.0131 | -0.0518 |
| R-squared | 0.438 | 0.341 | 0.485 |
| adjusted R-squared | 0.336 | 0.177 | 0.391 |

References

1. Al-Hebshi, N.N., et al., *Robust species taxonomy assignment algorithm for 16S rRNA NGS reads: application to oral carcinoma samples.* Journal of Oral Microbiology, 2015. **7**.
2. Guerrero-Preston, R., et al., *16S rRNA amplicon sequencing identifies microbiota associated with oral cancer, Human Papilloma Virus infection and surgical treatment.* Oncotarget, 2016. **7**(32): p. 51320-51334.
3. Hu, X.S., et al., *Changes in the salivary microbiota of oral leukoplakia and oral cancer.* Oral Oncology, 2016. **56**: p. E6-E8.
4. Lee, W.H., et al., *Bacterial alterations in salivary microbiota and their association in oral cancer.* Scientific Reports, 2017. **7**.
5. Hernandez, B.Y., et al., *Betel nut chewing, oral premalignant lesions, and the oral microbiome.* Plos One, 2017. **12**(2).
6. Bornigen, D., et al., *Alterations in oral bacterial communities are associated with risk factors for oral and oropharyngeal cancer.* Scientific Reports, 2017. **7**.
7. Wolf, A., et al., *The salivary microbiome as an indicator of carcinogenesis in patients with oropharyngeal squamous cell carcinoma: A pilot study.* Scientific Reports, 2017. **7**.
8. Zhao, H.S., et al., *Variations in oral microbiota associated with oral cancer.* Scientific Reports, 2017. **7**.
9. Al-hebshi, N.N., et al., *Inflammatory bacteriome featuring Fusobacterium nucleatum and Pseudomonas aeruginosa identified in association with oral squamous cell carcinoma.* Scientific Reports, 2017. **7**.
10. Amer, A., et al., *The Microbiome of Potentially Malignant Oral Leukoplakia Exhibits Enrichment for Fusobacterium, Leptotrichia, Campylobacter, and Rothia Species.* Frontiers in Microbiology, 2017. **8**.
11. Guerrero-Preston, R., et al., *High-resolution microbiome profiling uncovers Fusobacterium nucleatum, Lactobacillus gasseri/johnsonii, and Lactobacillus vaginalis associated to oral and oropharyngeal cancer in saliva from HPV positive and HPV negative patients treated with surgery and chemo-radiation.* Oncotarget, 2017. **8**(67): p. 110931-110948.
12. Shin, J.M., et al., *Microbial Communities Associated with Primary and Metastatic Head and Neck Squamous Cell Carcinoma - A High Fusobacterial and Low Streptococcal Signature.* Scientific Reports, 2017. **7**.
13. Wang, H.N., et al., *Microbiomic differences in tumor and paired-normal tissue in head and neck squamous cell carcinomas.* Genome Medicine, 2017. **9**.
14. Lim, Y., et al., *The Performance of an Oral Microbiome Biomarker Panel in Predicting Oral Cavity and Oropharyngeal Cancers.* Frontiers in Cellular and Infection Microbiology, 2018. **8**.
15. Perera, M., et al., *Inflammatory Bacteriome and Oral Squamous Cell Carcinoma.* Journal of Dental Research, 2018. **97**(6): p. 725-732.
16. Yang, C.Y., et al., *Oral Microbiota Community Dynamics Associated With Oral Squamous Cell Carcinoma Staging.* Frontiers in Microbiology, 2018. **9**.
17. Yang, S.F., et al., *Compositional and functional variations of oral microbiota associated with the mutational changes in oral cancer.* Oral Oncology, 2018. **77**: p. 1-8.
18. Hayes, R.B., et al., *Association of Oral Microbiome With Risk for Incident Head and Neck Squamous Cell Cancer.* Jama Oncology, 2018. **4**(3): p. 358-365.

19. Hsiao, J.R., et al., *The interplay between oral microbiome, lifestyle factors and genetic polymorphisms in the risk of oral squamous cell carcinoma.* Carcinogenesis, 2018. **39**(6): p. 778-787.
20. Zhang, Z., et al., *Compositional and Functional Analysis of the Microbiome in Tissue and Saliva of Oral Squamous Cell Carcinoma.* Frontiers in Microbiology, 2019. **10**.
21. Hashimoto, K., et al., *Changes in oral microbial profiles associated with oral squamous cell carcinoma vs leukoplakia.* J Investig Clin Dent, 2019. **10**(4): p. e12445.
22. Ganly, I., et al., *Periodontal pathogens are a risk factor of oral cavity squamous cell carcinoma, independent of tobacco and alcohol and human papillomavirus.* International Journal of Cancer, 2019. **145**(3): p. 775-784.
23. Zhang, L., et al., *The Oral Microbiota May Have Influence on Oral Cancer.* Frontiers in Cellular and Infection Microbiology, 2020. **9**.
